# Supplementary material for: Machine learning assisted single-molecule sensing towards standard-free quantification of per- and polyfluoroalkyl carboxylic acids
Source: Nat Commun. 2026 Mar 13;17:3923. doi: 10.1038/s41467-026-70718-3 (PMC13128836; doi:10.1038/s41467-026-70718-3)
Supplement: Supplementary file 1 — Supplemantary Information [file 41467_2026_70718_MOESM1_ESM.pdf]

Supplementary Information

Machine learning assisted single-molecule sensing towards standard-free quantification of per- and polyfluoroalkyl carboxylic acids

Jiaqi Zuo[#], Hong-Shuang Li[#], Wen Tang[#], Xian Zhao, Meng-Yuan Cheng, Zekai Yang,

Siyu Tian, Pufeng Li, Xueying Xie, Dan Luo, & Kaipei Qiu[*]

I Supplementary methods 1
i Construction of feature library with known PFCA samples of high purity.............................1
ii Expansion of feature library with other PFCA samples in real samples 2
iii Simultaneous quantification theory under different interferences 3
II Standard-free prediction 5
i Chemical information of all PFCA analytes 5
ii Per-fluoroalkyl carboxylic acids 7
iii Poly-fluoroalkyl carboxylic acids substituted by H- / Cl- groups.......................................10
iv Poly-fluoroalkyl carboxylic acids substituted by other groups 12
v Assessment of volume-current relationship under various salt concentrations 13
III Standard-free identification 14
i Feature extraction of PFCA analytes and R_6 probe..14
ii Rank of feature importances 28
iii Classification performances of different classifiers 38
iv Rank of feature importances with different dataset sizes 39
v Clustering to differentiate PFCA and FA of same blockade..42
IV Standard-free quantification 43
i Capture rates for linear PFCAs of different chain lengths 43
ii Standard-free quantification of short-chain PFCAs 44
iii Simultaneous quantification ability in mixed analytes...45
iv Robust quantification ability under various interferences...50
v Strong quantification ability with low limit of detection 54
V Optimization and applications..56
i Consistent identification performance under various quantification conditions 56
ii Energy barrier regulation towards standard-free quantification...59
iii Blockade prediction and identification performance of PFCA isomers..............................60
iv Reliability of the interval time calculation 63
v Incubation of PFCAs with R_6 probes 64
VI Supplementary references..65

I Supplementary methods

i Construction of feature library with known PFCA samples of high purity

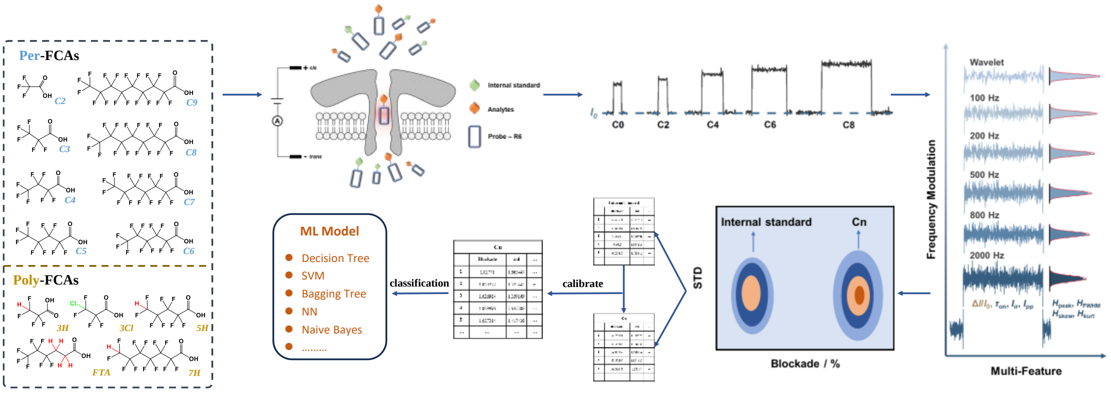

Supplementary Method 1. Process of data acquisition, feature extraction and model training with known PFCAs.

During the initial construction process of multi-dimensional feature library with known PFCAs (commercial standard is NOT required), the following methodology should be employed:

Step a, Performing nanopore single-molecule measurements and analysis for individual known PFCAs in the presence of internal standards to acquire raw current signals;

Step b, Extracting the frequency-modulated multi-dimensional features for target PFCAs and internal standards using customized script;

Step c, Applying the two-dimensional kernel density plot analysis to primarily differentiate the signals of target PFCAs from internal standards;

Step d, Calibrating the multi-dimensional features of target PFCAs against internal standards;

Step e, Using those labeled multi-dimensional features of target PFCAs to train and select the appropriate classification models.

Notes: Multiple experiments were performed for all analytes mentioned in the manuscript to obtain at least three sets of valid parallel experimental data. All of the obtained data were aggregated and labeled. For any given PFCA, 2000 events were randomly selected (totally 28000), of which 80% were randomly selected for model training and the remaining 20% were used for testing.

ii Expansion of feature library with other PFCA samples in real samples

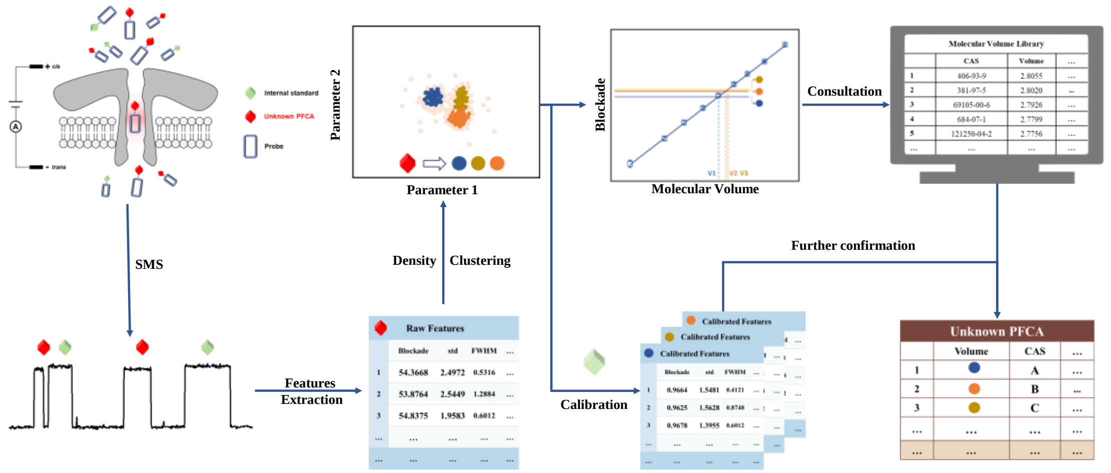

Supplementary Method 2. Process of standard-free identification and feature extraction for unknown PFCAs in real samples.

When the known high-purity PFCA sample is not available, it is also possible to further expand feature library with real samples containing other PFCAs of an approximately known concentration, e.g., chemical raw materials, industrial by-products, degradation products, et al. To achieve this, the following methodology should be employed:

Step a, Performing nanopore single-molecule measurements and analysis for real samples with unknown PFCAs in the presence of internal standards to acquire raw current signals;

Step b, Extracting the frequency-modulated multi-dimensional features of the potential signals for unknown PFCAs and internal standards using customized script;

Step c, Applying density-based spatial clustering algorithms to differentiate the signal features of unknown PFCAs and internal standards, and to primarily confirm the identity of unknown PFCAs based on the signal ratios of individual clusters and their approximate abundance in real samples;

Step d, Calculating the current blockade of the cluster events induced by the unknown PFCAs, and utilizing the linear volume-blockade relationship to estimate their respective molecular volumes, as well as using internal standards to calibrate current signals and to reduce experimental errors. The identity of unknown PFCAs is double confirmed with the MD simulated molecular volume library;

Step e, Collecting and labeling the current signals induced by those identified unknown PFCAs, extracting the respective multi-dimensional features and including them into the feature library.

Notes: According to the confidence on the pre-obtained abundance for unknown PFCAs in real samples, the order of steps c and d could be switched in particular applications.

iii Simultaneous quantification theory under different interferences

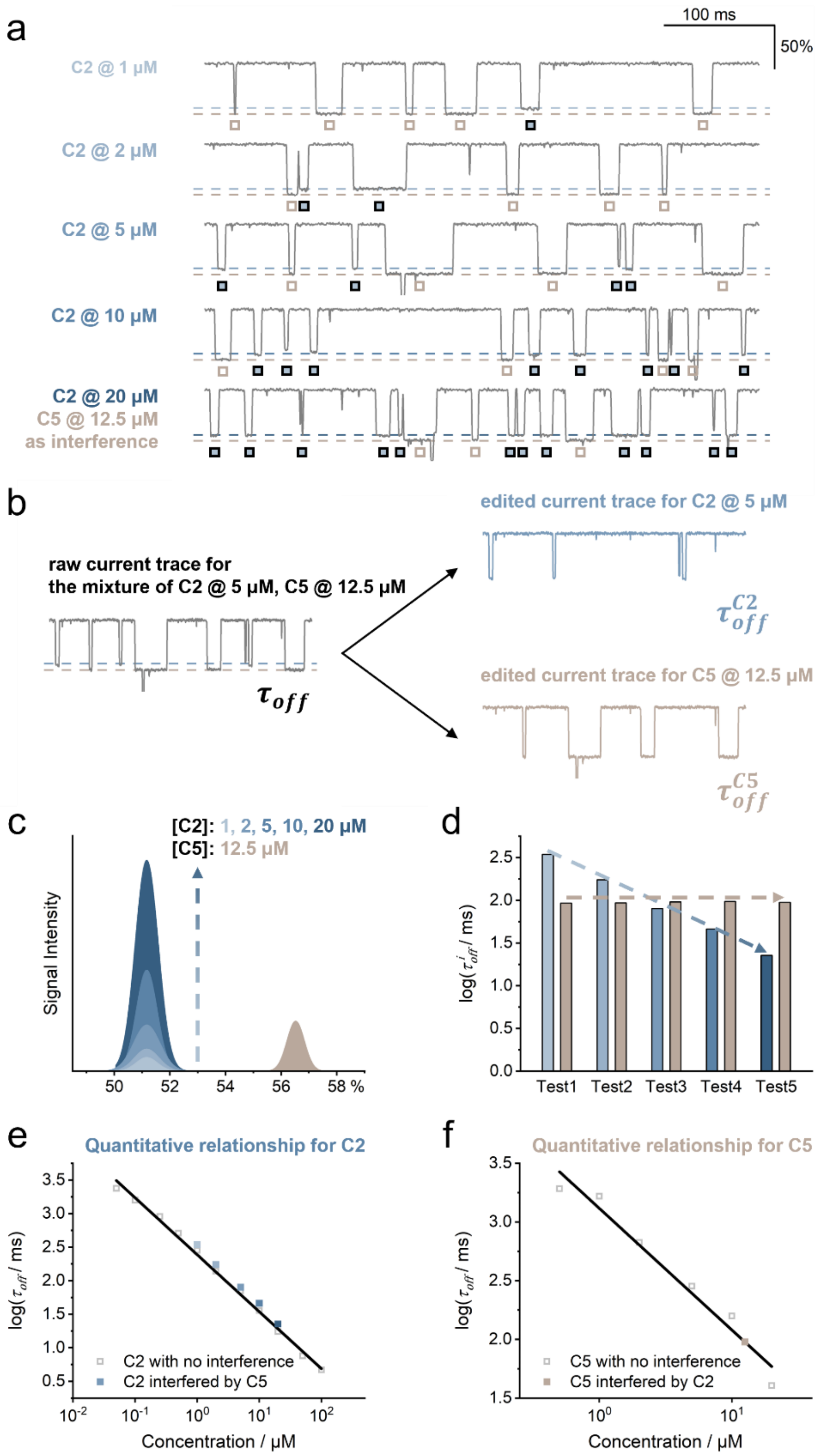

Supplementary Method 3. Simultaneous quantification of multiple PFCAs.

Simultaneous quantification of specific components in mixed analytes adopts the well-established method proposed by Hagan Bayley[1,2]. In this work, sequential addition experiment of C2-R_6 under the interference of C5-R_6 was set as an example to demonstrate the simultaneous quantification of C2-R_6 as well as C5-R_6.

Step a, Recording the raw current traces of C2-R_6 under different concentrations (1/2/5/10/20

μM) interfered by C5-R_6 (12.5 μM). Herein, blue squares represent current blockade events caused by C2, while brown ones represent C5-caused current blockade events;

Step b, Illustration of how to create edited current traces for C2 and C5 individually from the raw current trace recording single-molecule signals caused by both C2 and C5. Notably, the overall interval time (τ_{off}) can be directly measured from the raw current trace, while the specific-component interval time (τ_{off}^{i}) can be calculated and extracted from the edited current trace for C2 or C5. Further, the τ_{off}^{i} refers to the actual interval time produced by one certain component, eliminating the impact of any other interferences;

Step c, Applying the frequency-modulated multi-feature classification to obtain the accurate numbers of C2 and C5 signals, individually;

Step d, Calculating the τ_{off}^{i} according to the signal numbers of C2 and C5. Calculation formula for the specific-component interval time (τ_{off}^{i}) is as follows:

$$\tau_{off}^{i} = \tau_{off} \times \frac{\sum_{1}^{m} N_i}{N_i},$$

where m denotes the total number of all components in mixed analytes, and N_i denotes the signal number of component i;

Step e & f, The concentration of component i in mixed analytes can be calculated with the τ_{off}^{i}, through the quantitative relationship for C2-R_6 or C5-R_6, individually.

Notes: Theoretically, the calculation formula in **step d** can be extrapolated to quantification under more than one interference provided the total signal number and the signal number for specific components are obtained.

II Standard-free prediction

i Chemical information of all PFCA analytes

Supplementary Table 1. Information of all PFCA analytes involved in this manuscript

Substance	Molecular Formula	CAS number	Abbreviation /Label
Trifluoroacetic acid	$C_2HF_3O_2$	76-05-1	C2
Pentafluoropropionic acid	$C_3HF_5O_2$	422-64-0	C3
Heptafluorobutyric acid	$C_4HF_7O_2$	375-22-4	C4
Perfluorovaleric acid	$C_5HF_9O_2$	2706-90-3	C5
Perfluorohexanoic acid	$C_6HF_{11}O_2$	307-24-4	C6
Perfluoroheptanoic acid	$C_7HF_{13}O_2$	375-85-9	C7
Perfluorooctanoic acid	$C_8HF_{15}O_2$	335-67-1	C8
Perfluorononanoic acid	$C_9HF_{17}O_2$	375-95-1	C9
Perfluorododecanoic acid	$C_{12}HF_{23}O_2$	307-55-1	C12
Perfluorotetradecanoic acid	$C_{14}HF_{27}O_2$	376-06-7	C14
3H-Tetrafluoropropionic acid	$C_3H_2F_4O_2$	756-09-2	3H
3Cl-Tetrafluoropropionic acid	$C_3HClF_4O_2$	661-82-5	3Cl
5H-Octafluoropentanoic acid	$C_5H_2F_8O_2$	376-72-7	5H
3:3 Fluorotelomer carboxylic acid	$C_6H_5F_7O_2$	356-02-5	FTA
7H-Dodecafluoroheptanoic acid	$C_7H_2F_{12}O_2$	1546-95-8	7H
(R)-3,3,3-Trifluoro-2-hydroxypropanoic acid	$C_3H_3F_3O_3$	121250-04-2	2OH-D
3,3,3-Trifluoro-2-methylpropanoic acid	$C_4H_5F_3O_2$	381-97-5	SYN28
3-(Trifluoromethyl)crotonic acid	$C_5H_5F_3O_2$	69056-67-3	33
2,3,4,5-Tetrafluorobenzoic acid	$C_7H_2F_4O_2$	1201-31-6	A2345
2,3,4,6-Tetrafluorobenzoic acid	$C_7H_2F_4O_2$	32890-92-9	A2346
2,3,5,6-Tetrafluorobenzoic acid	$C_7H_2F_4O_2$	652-18-6	A2356
2,4-Bis(trifluoromethyl)benzoic acid	$C_9H_4F_6O_2$	32890-87-2	B24
2,5-Bis(trifluoromethyl)benzoic acid	$C_9H_4F_6O_2$	42580-42-7	B25

2,6-Bis(trifluoromethyl)benzoic acid	$C_9H_4F_6O_2$	24821-22-5	B26
3,4-Bis(trifluoromethyl)benzoic acid	$C_9H_4F_6O_2$	133804-66-7	B34
3,5-Bis(trifluoromethyl)benzoic acid	$C_9H_4F_6O_2$	725-89-3	B35

ii Per-fluoroalkyl carboxylic acids

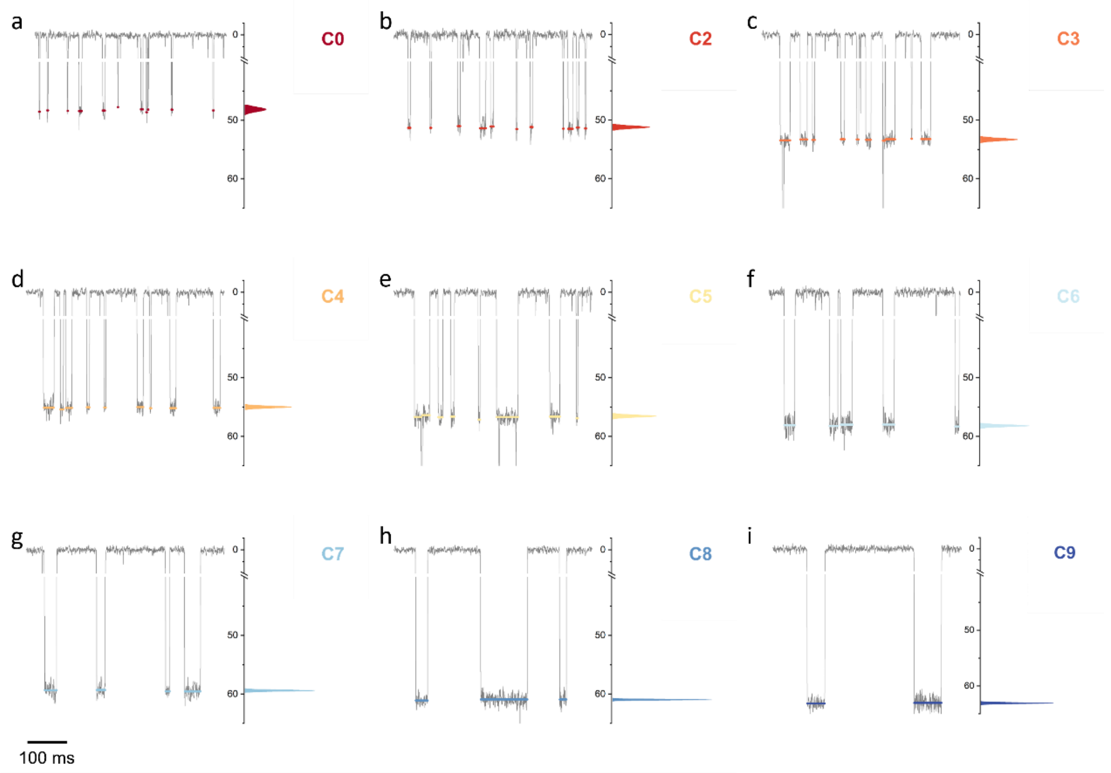

Supplementary Fig. 1. Representative fragments of current traces (left) and histograms of current blockades (right) induced by C0- to C9-R_6 conjugates with the applied voltage of -50 mV.

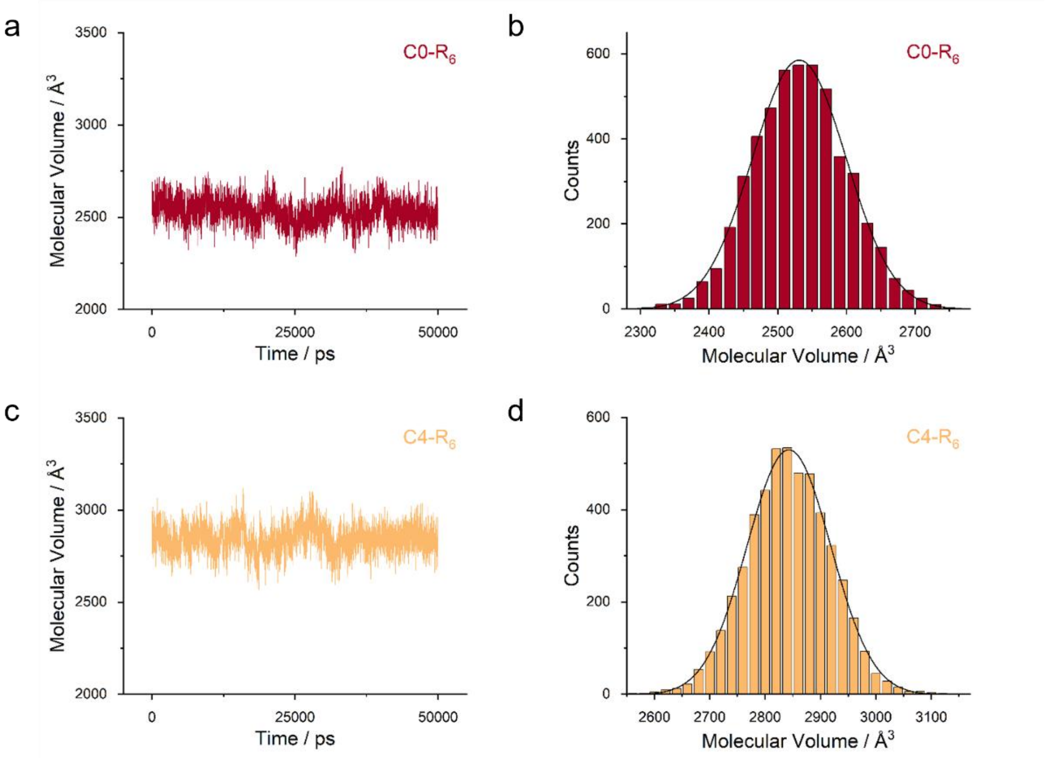

Supplementary Fig. 2. (a, c) Simulation results of molecular volume of C0/C4-R_6; (b, d) Histograms of molecular volume of C0/C4-R_6.

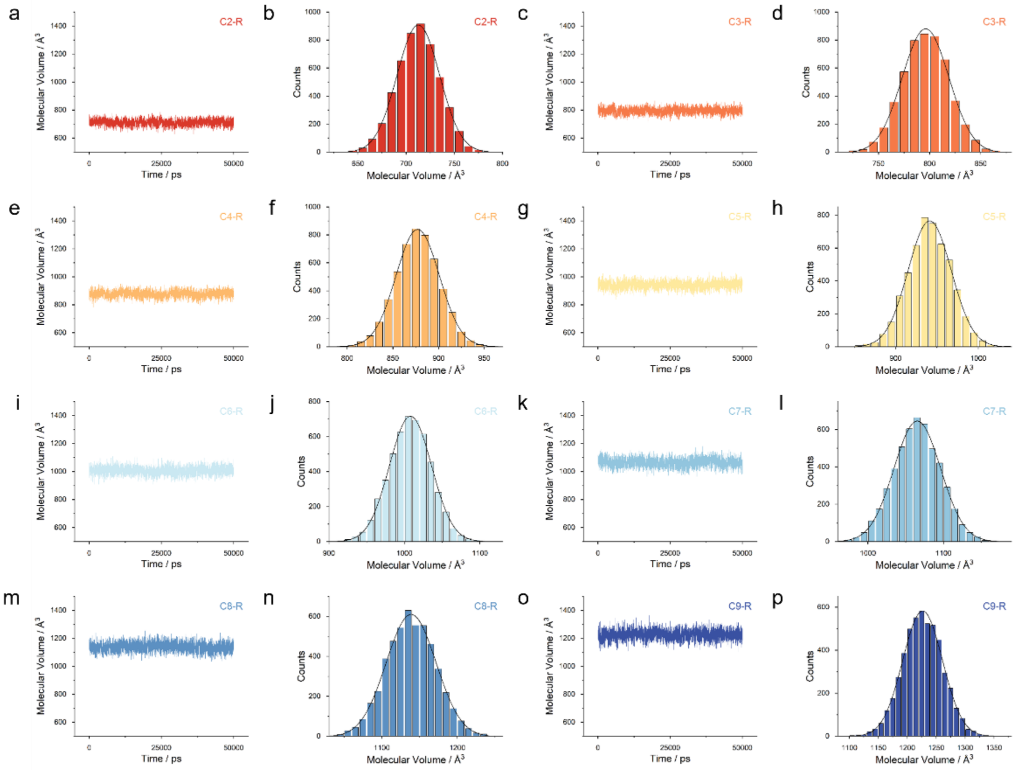

Supplementary Fig. 3. Simulation results and histograms of molecular volume induced by the C2- to C9-R conjugates.

iii Poly-fluoroalkyl carboxylic acids substituted by H- / Cl- groups

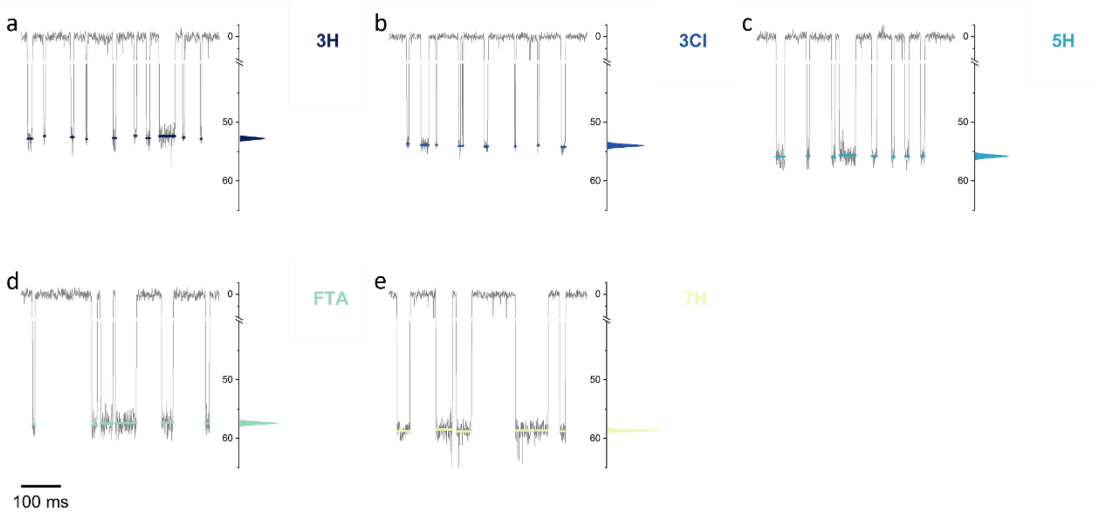

Supplementary Fig. 4. Representative fragments of current traces (left) and histograms of current blockades (right) induced by 3H/3Cl/5H/FTA/7H-R_6 conjugates with the applied voltage of -50 mV.

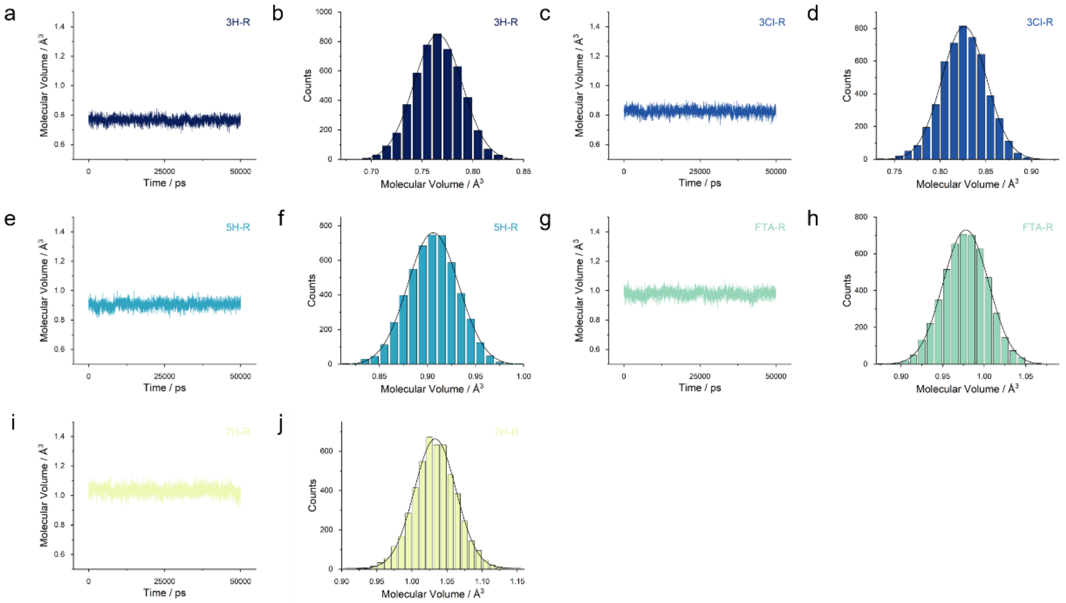

Supplementary Fig. 5. Simulation results and histograms of molecular volume induced by 3H/3Cl/5H/FTA/7H-R conjugates.

iv Poly-fluoroalkyl carboxylic acids substituted by other groups

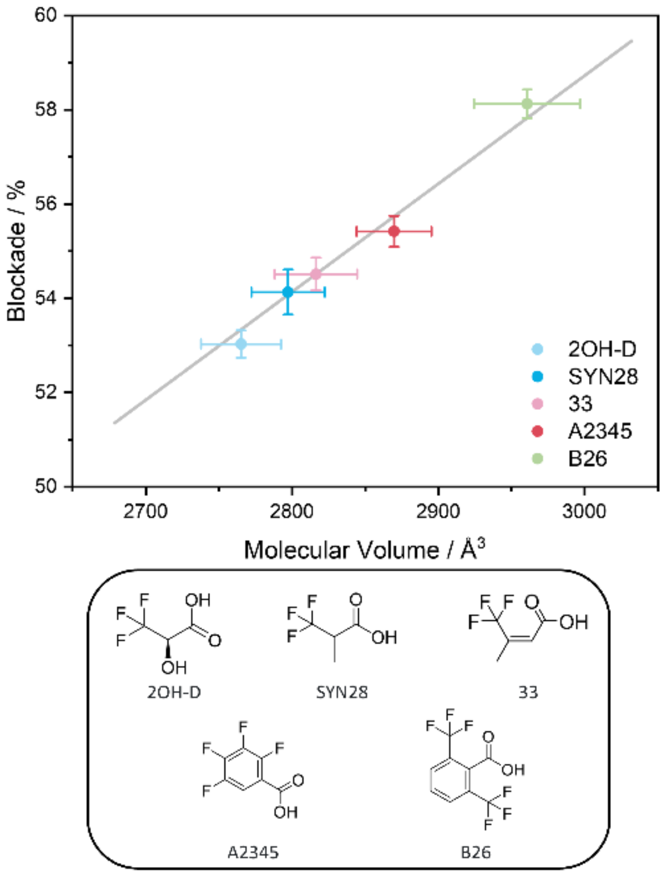

Supplementary Fig. 6. Experimentally measured current blockade and molecular volume of poly-fluoroalkyl carboxylic acids substituted by other groups, along with the established volume-current relationship (grey line). The error bars for x-axis were standard deviations calculated from three parallel molecular volume simulations. The error bars for y-axis were standard deviations obtained from the histograms of current blockades.

v Assessment of volume-current relationship under various salt concentrations

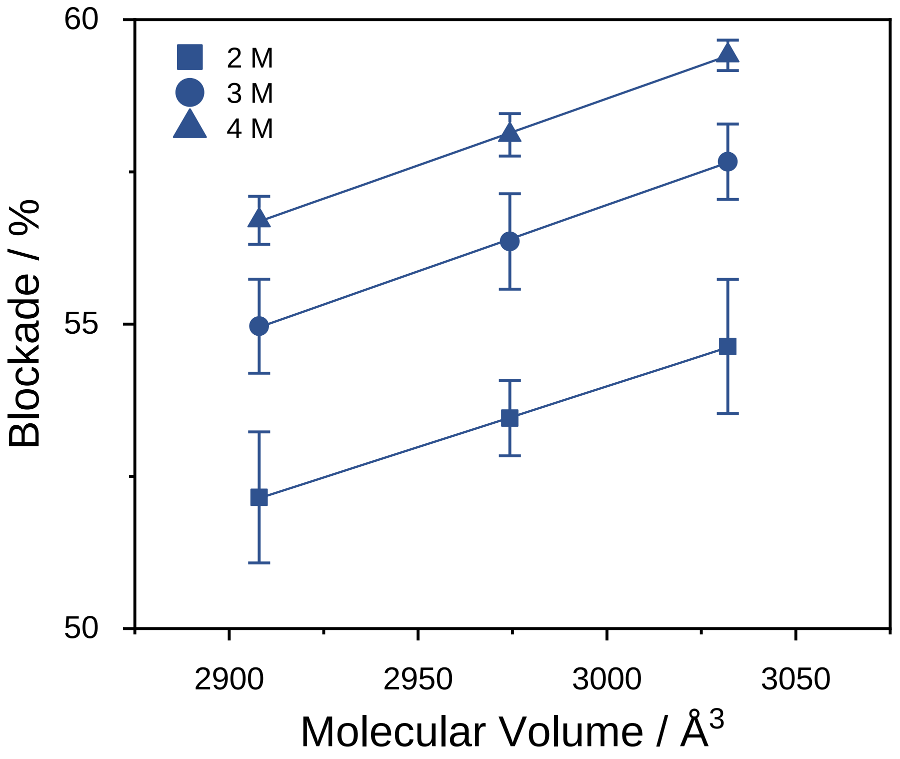

Supplementary Fig. 7. The linear fitting results of C5-, C6- and C7-R_6 in 2/3/4 M KCl (linear fit, R^2 = 0.9995-0.9999). The error bars were two times standard deviations obtained from the histograms of current blockades of C5-, C6- and C7-R_6 in 2/3/4 M KCl.

III Standard-free identification

i Feature extraction of PFCA analytes and R_6 probe

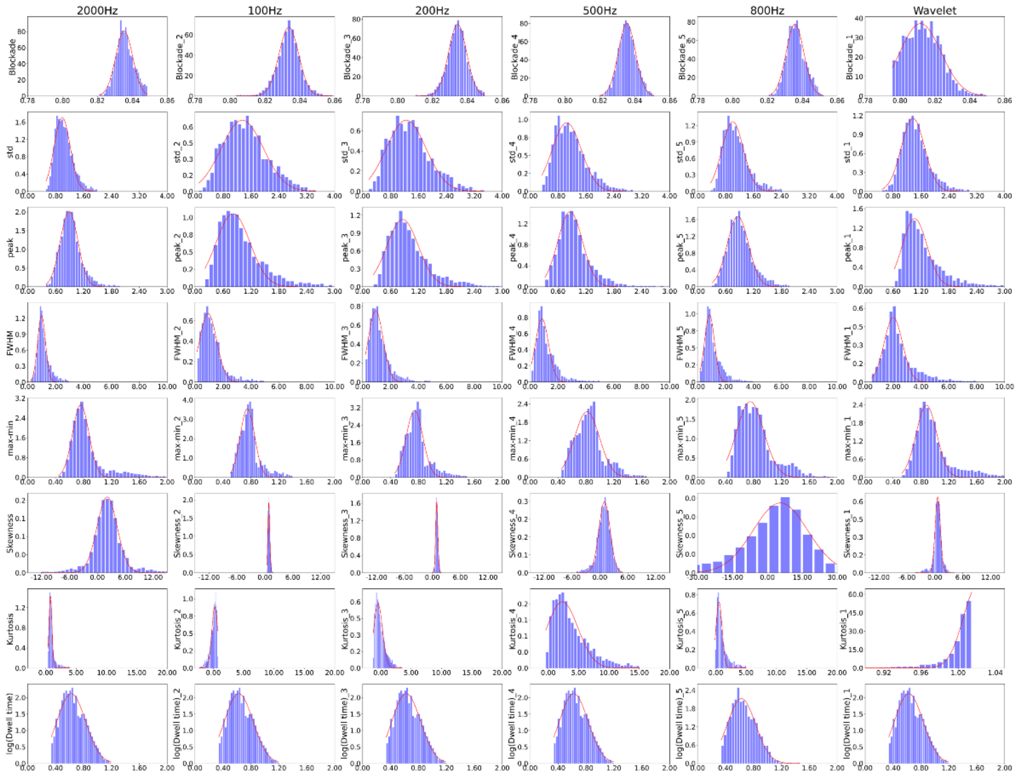

Supplementary Fig. 8. Histograms of 43 features of C0.

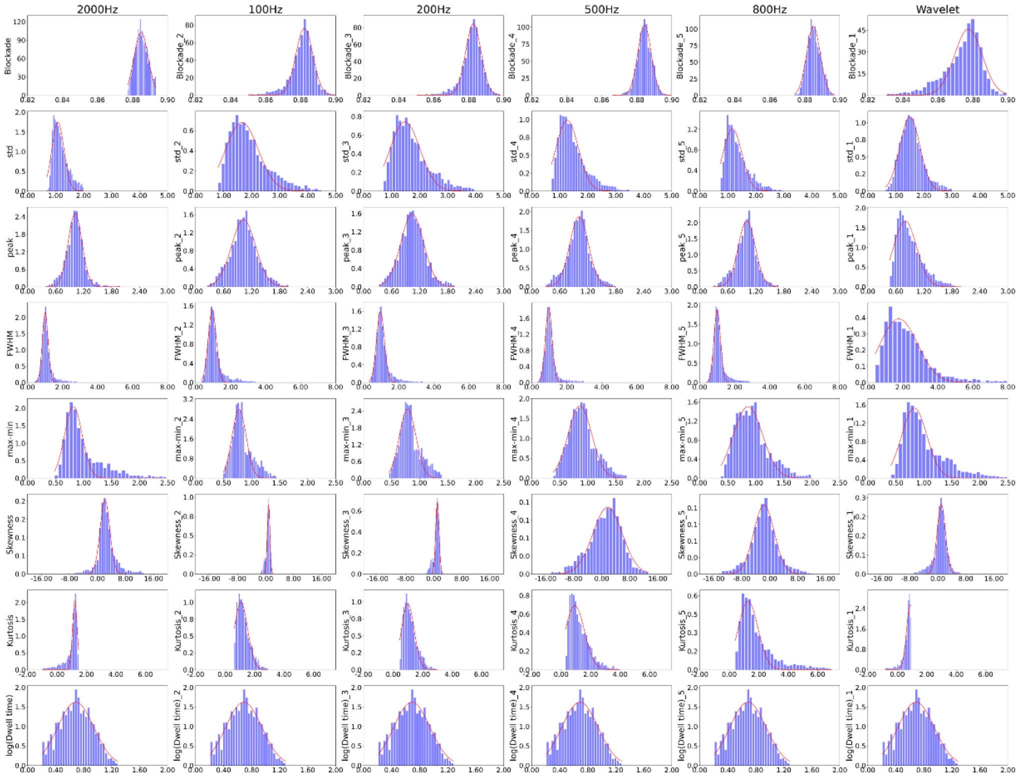

Supplementary Fig. 9. Histograms of 43 features of C2.

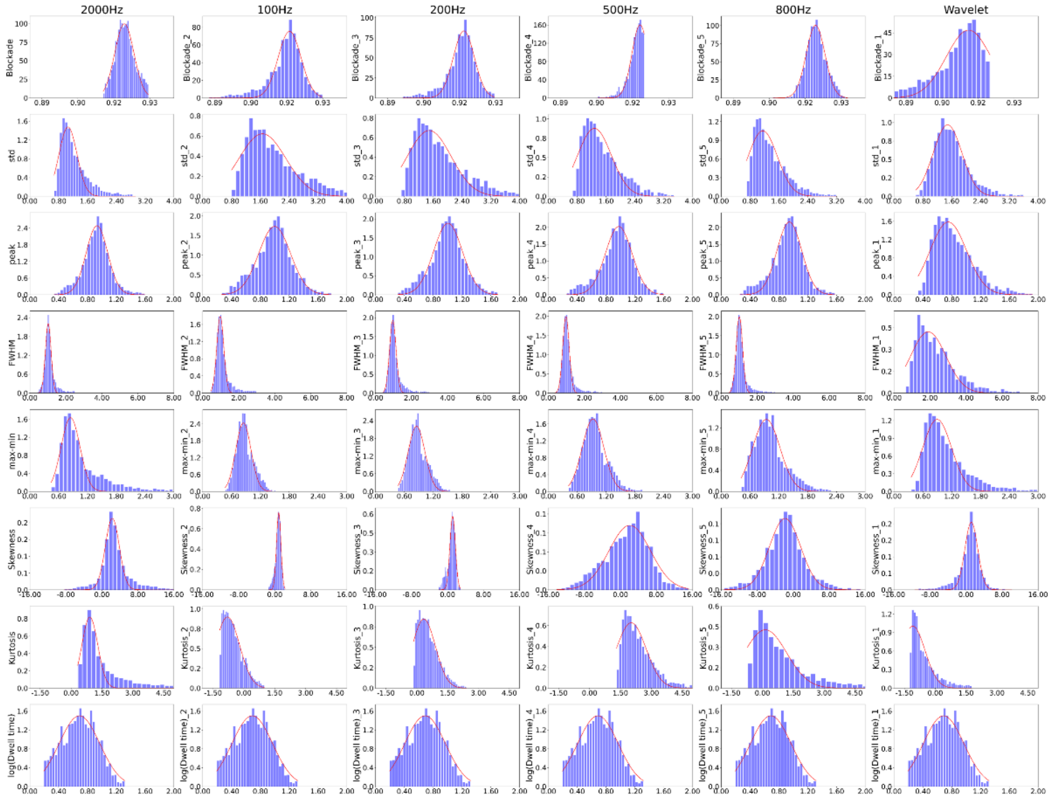

Supplementary Fig. 10. Histograms of 43 features of C3.

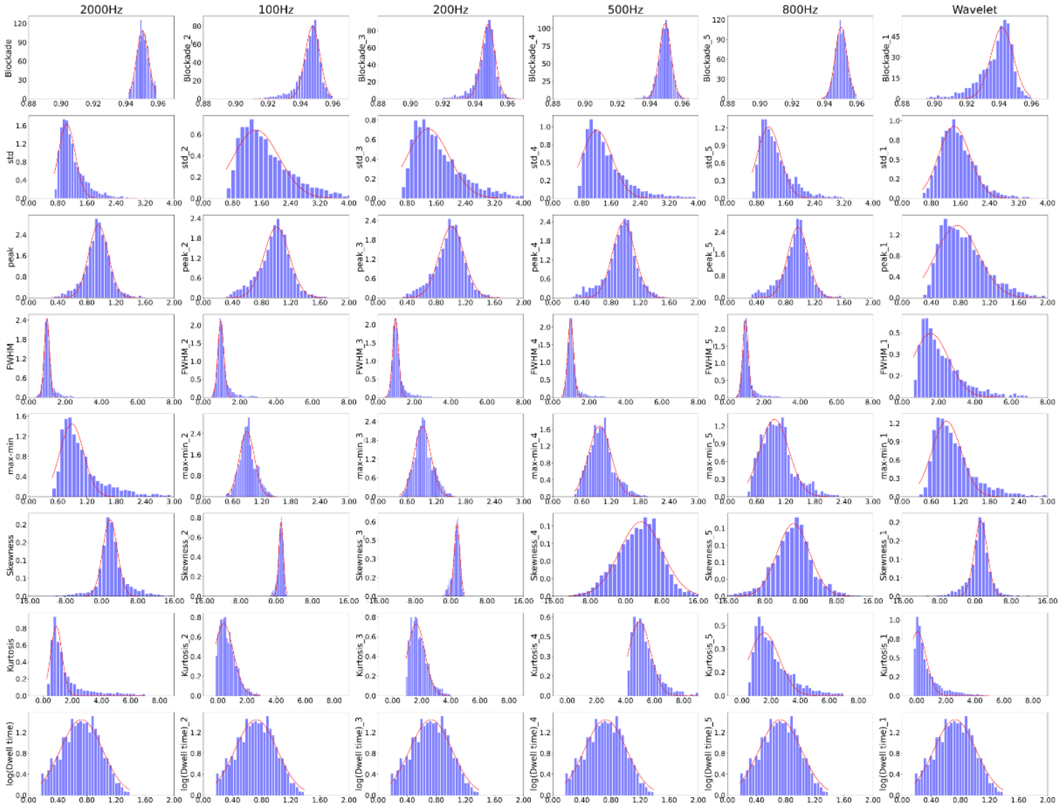

Supplementary Fig. 11. Histograms of 43 features of C4.

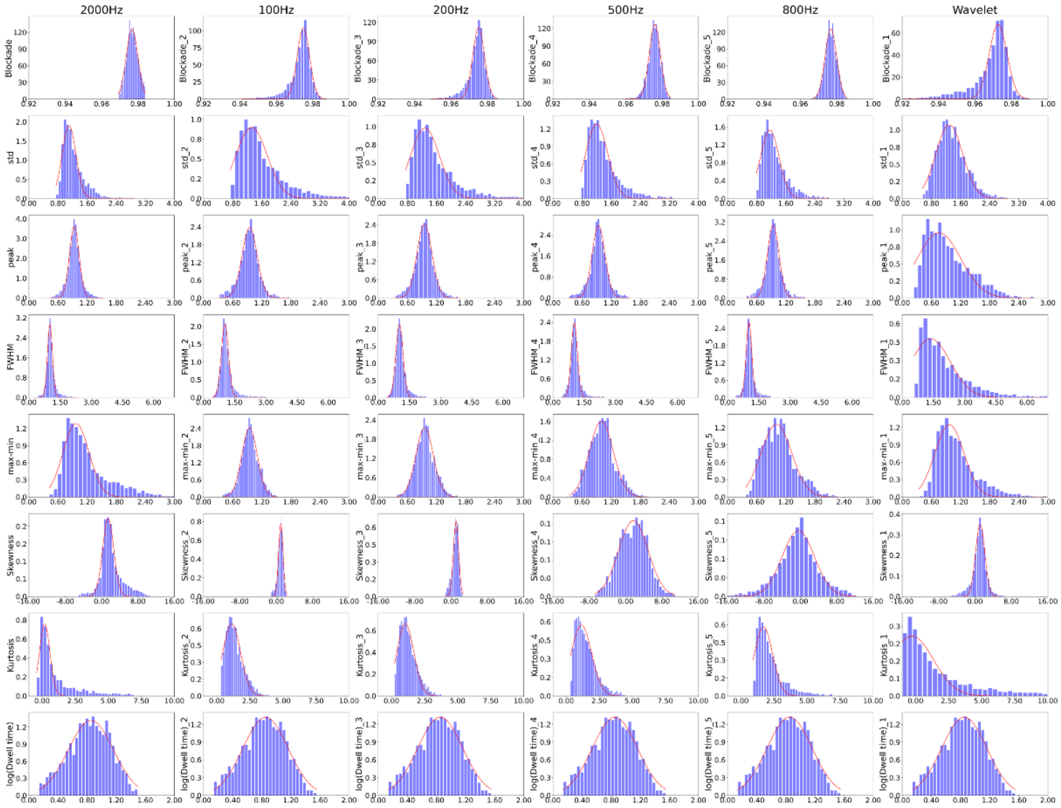

Supplementary Fig. 12. Histograms of 43 features of C5.

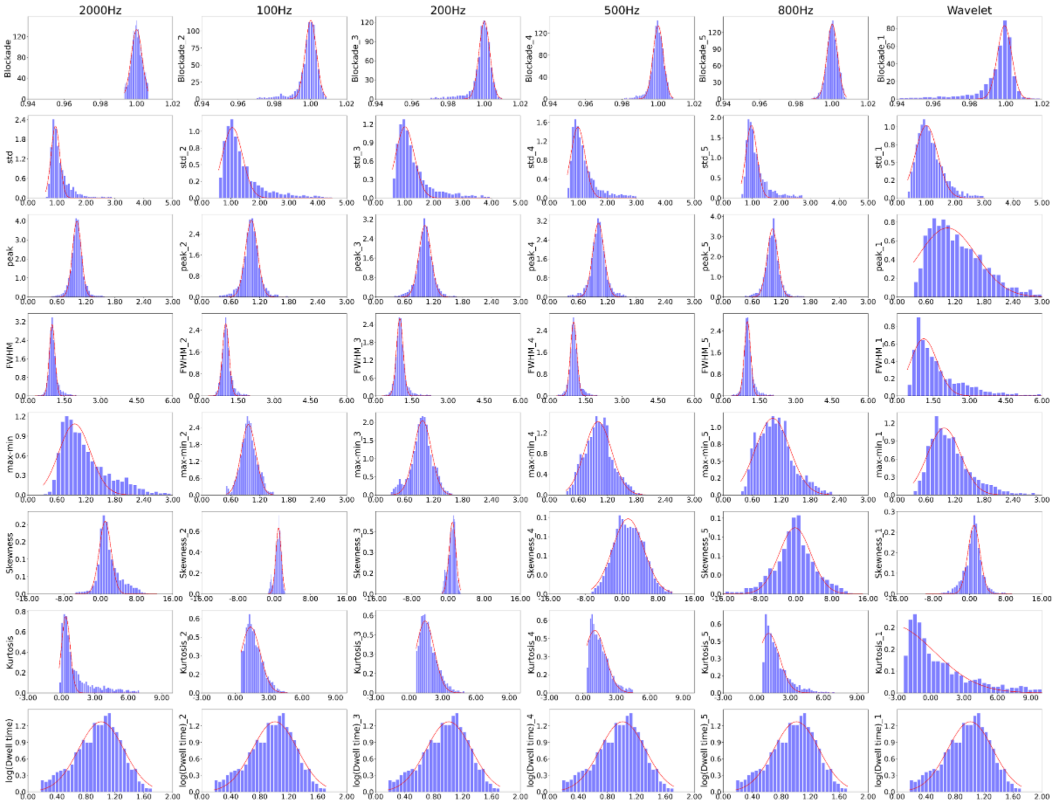

Supplementary Fig. 13. Histograms of 43 features of C6.

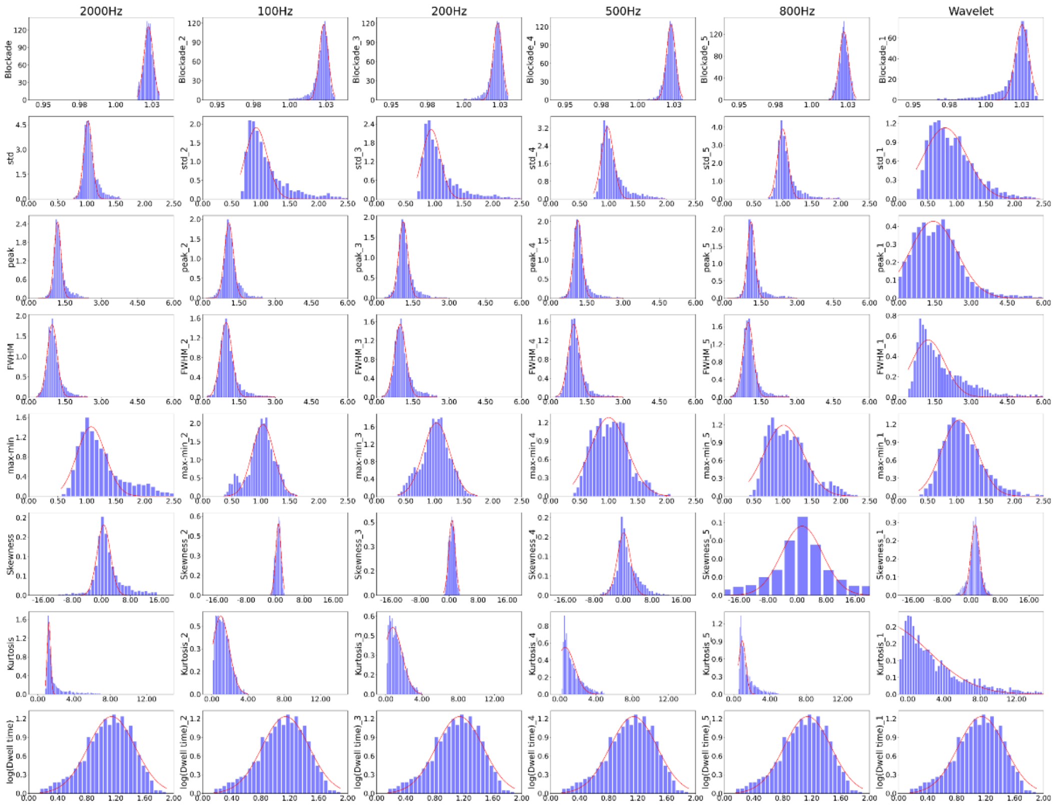

Supplementary Fig. 14. Histograms of 43 features of C7.

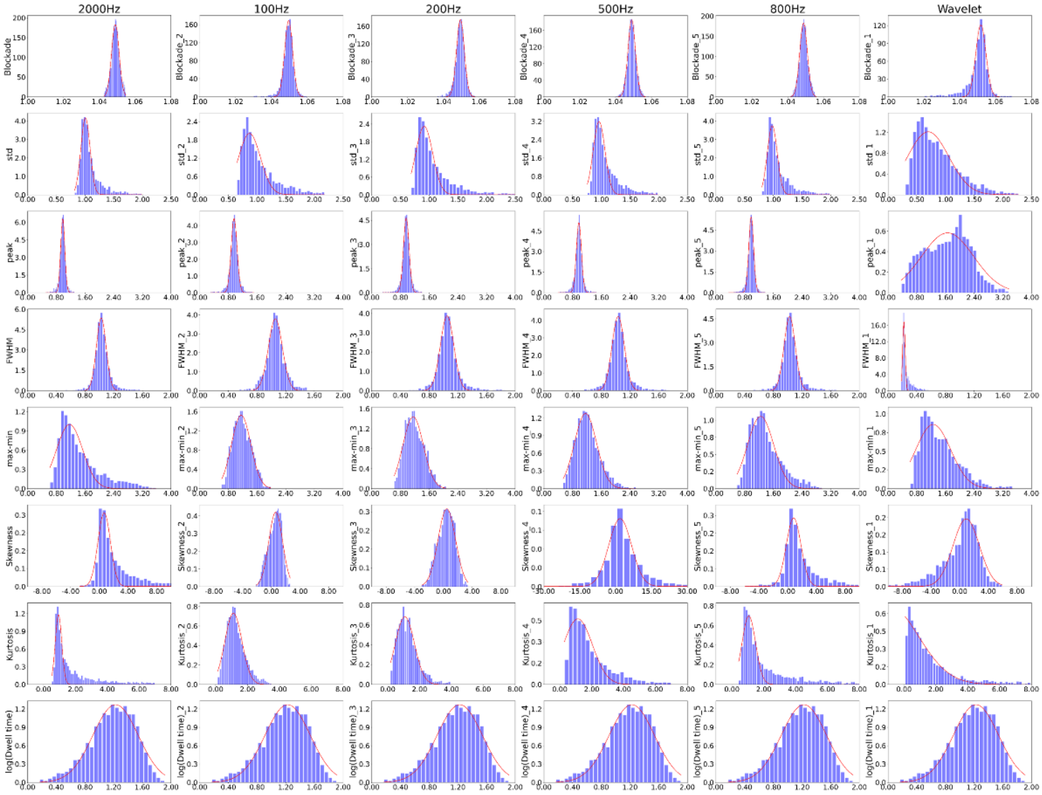

Supplementary Fig. 15. Histograms of 43 features of C8.

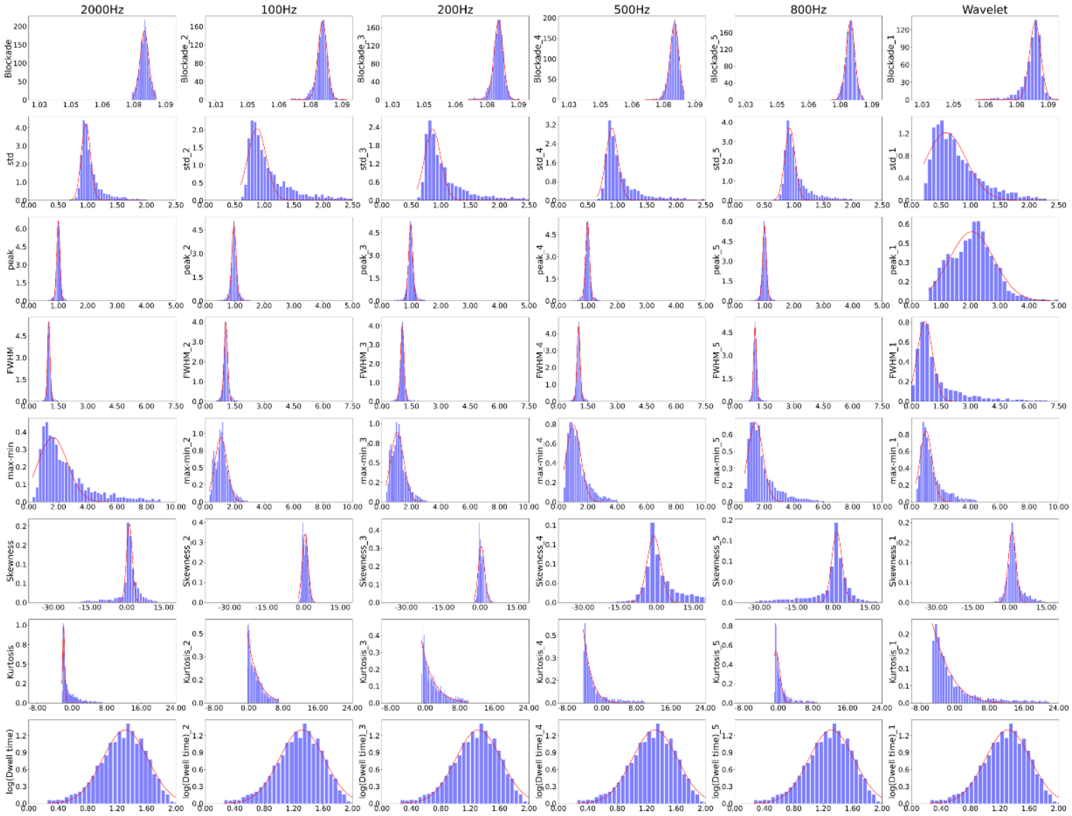

Supplementary Fig. 16. Histograms of 43 features of C9.

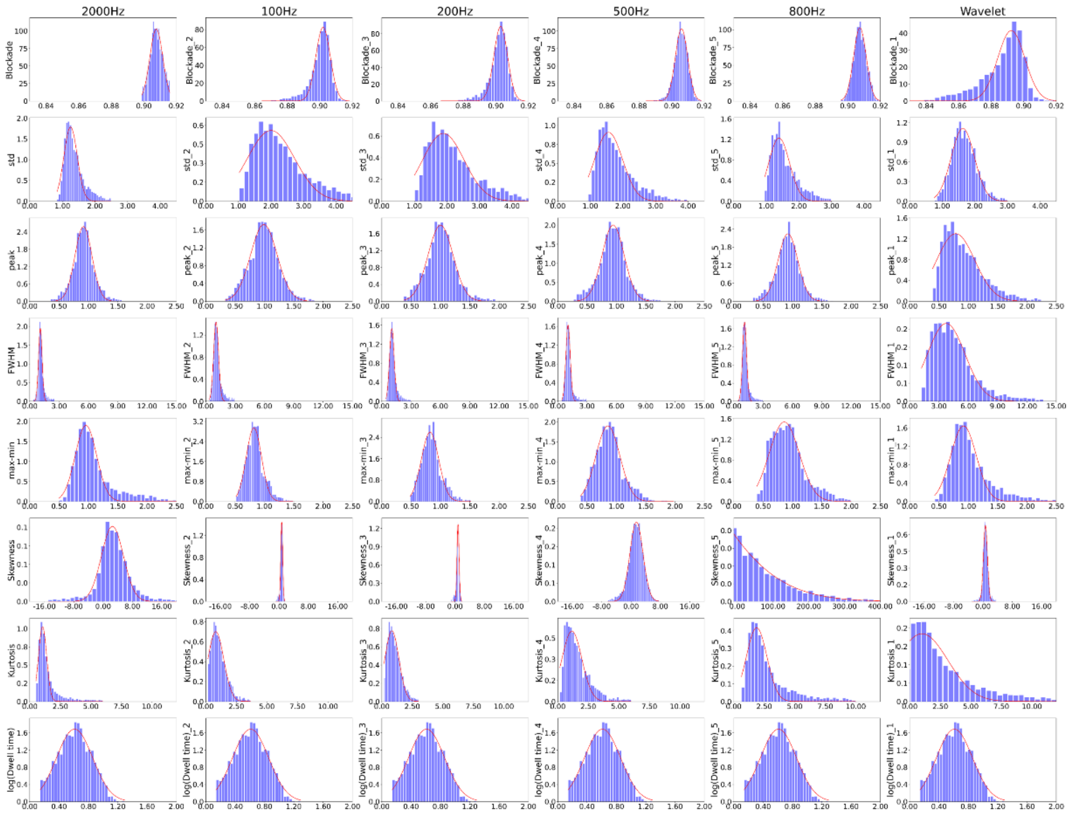

Supplementary Fig. 17. Histograms of 43 features of 3H.

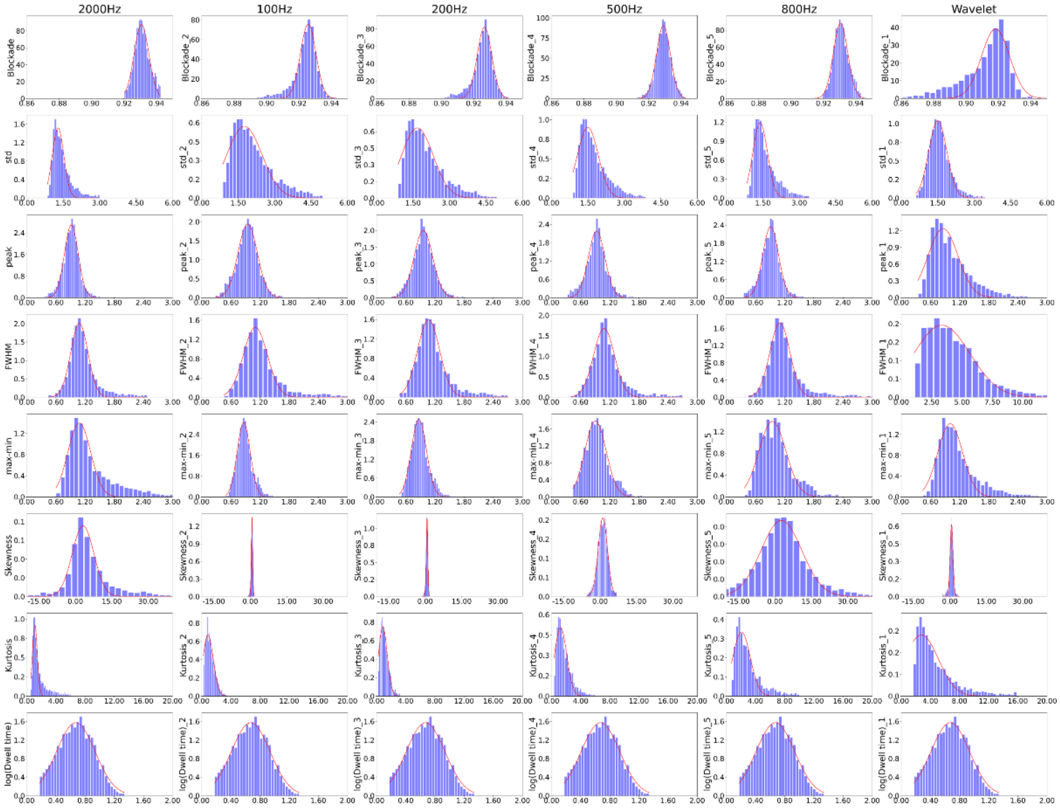

Supplementary Fig. 18. Histograms of 43 features of 3Cl.

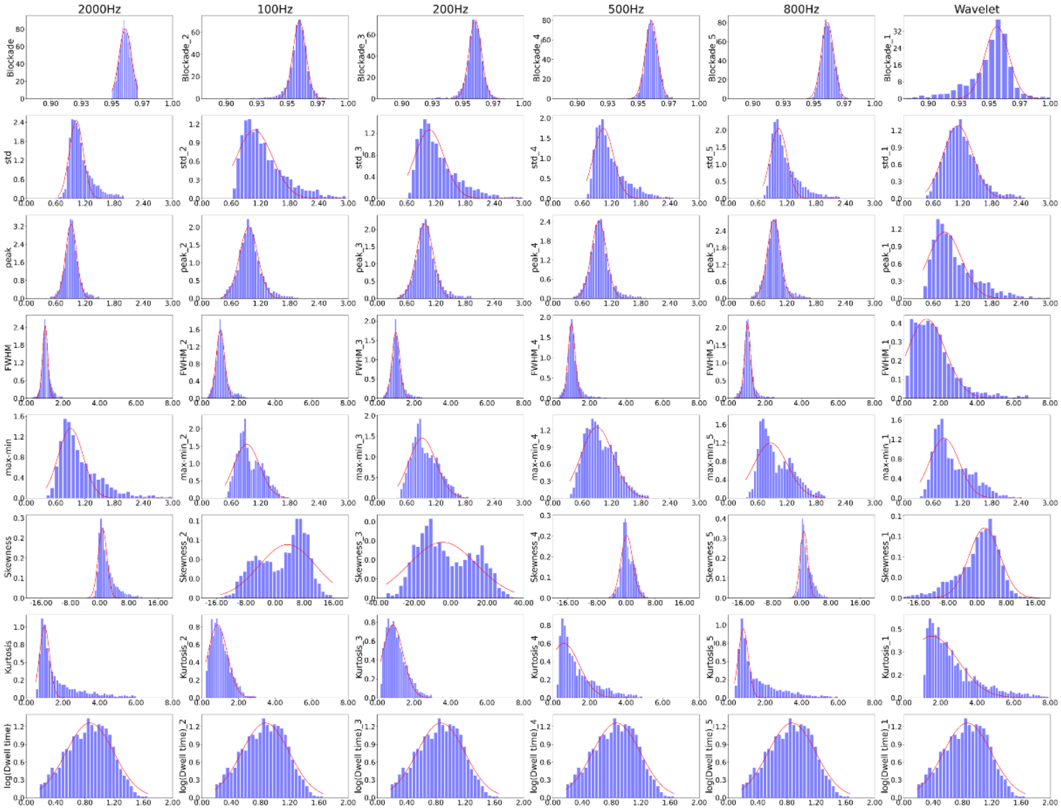

Supplementary Fig. 19. Histograms of 43 features of 5H.

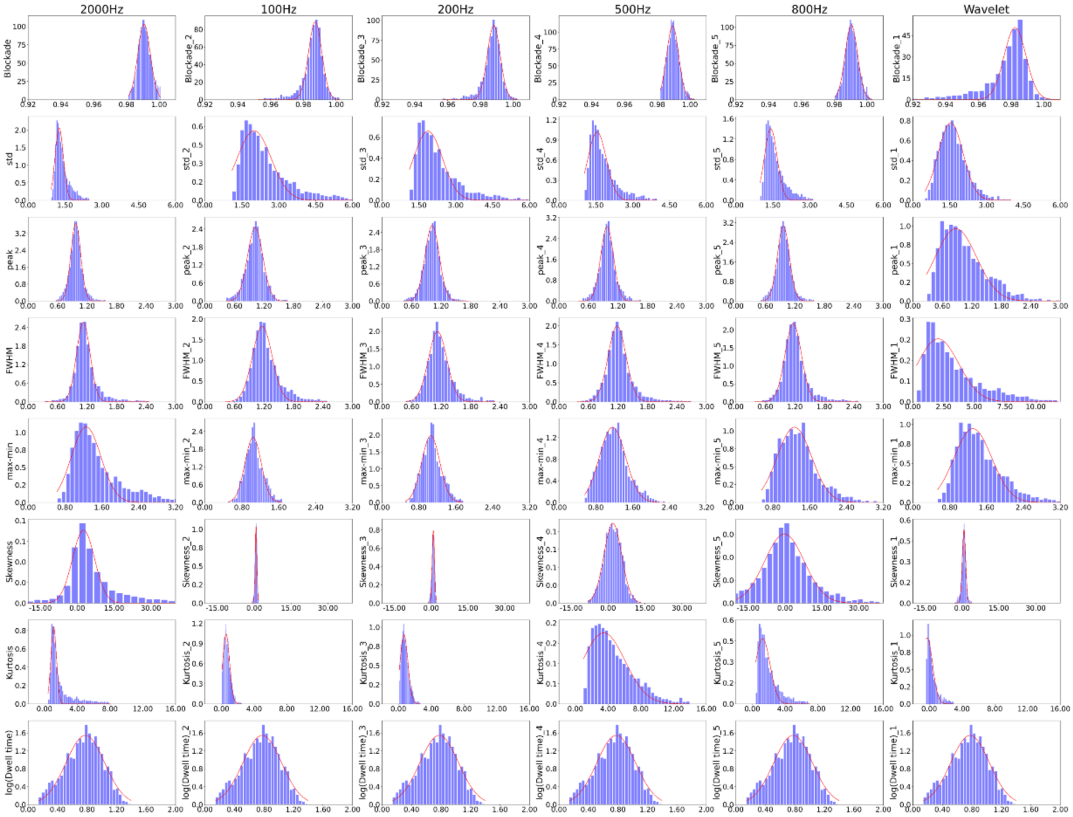

Supplementary Fig. 20. Histograms of 43 features of FTA.

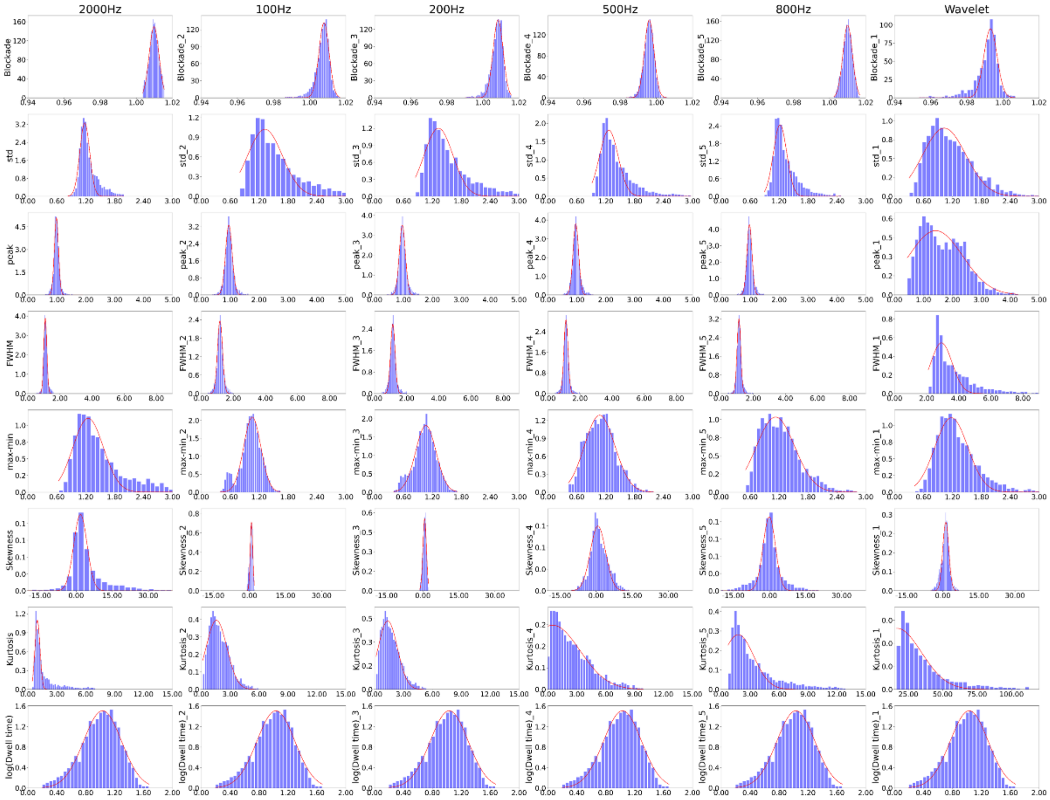

Supplementary Fig. 21. Histograms of 43 features of 7H.

ii Rank of feature importances

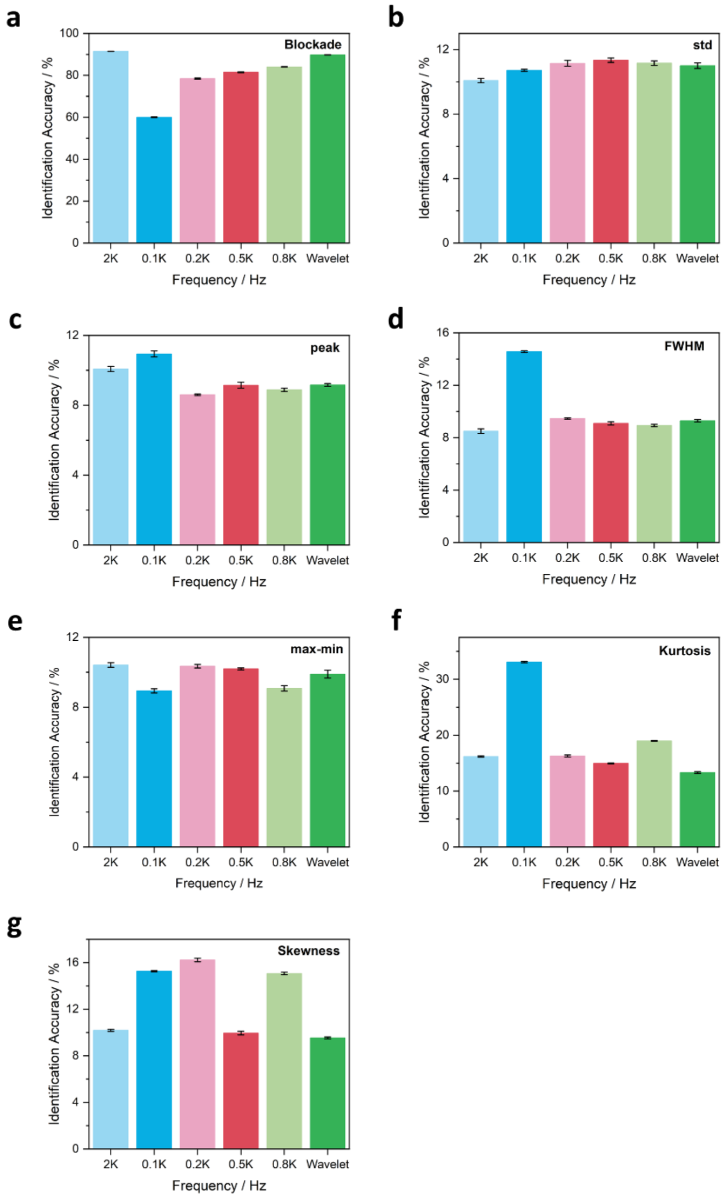

Supplementary Fig. 22. The identification accuracy of 14 analytes in single feature dimension. The error bars were standard deviations calculated from five parallel classifications.

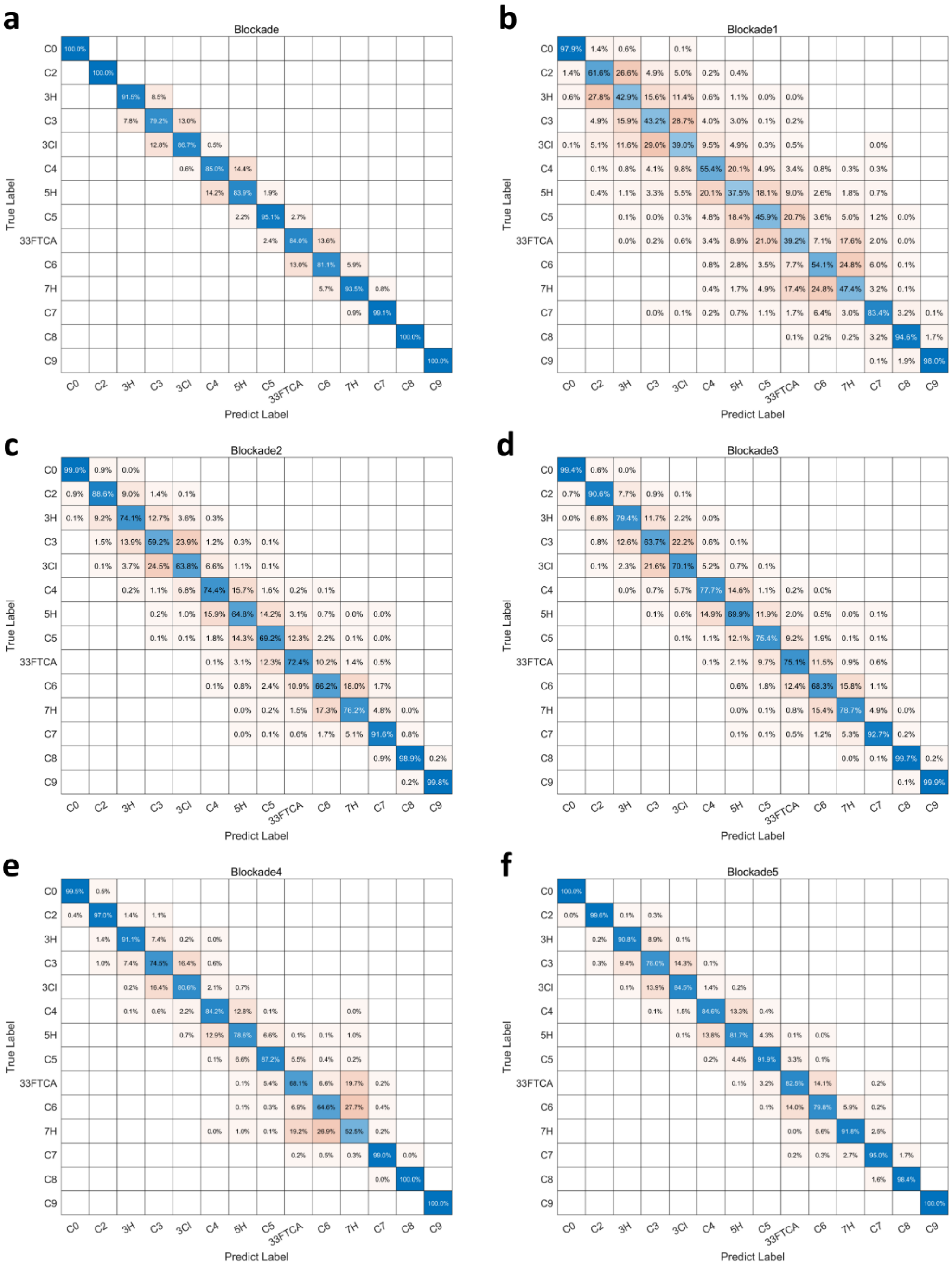

Supplementary Fig. 23. The confusion matrix of 14 analytes in $\Delta I/I_0$ dimension, (a) original signal, (b) wavelet, (c) 100Hz, (d) 200Hz, (e) 500Hz, (f) 800Hz.

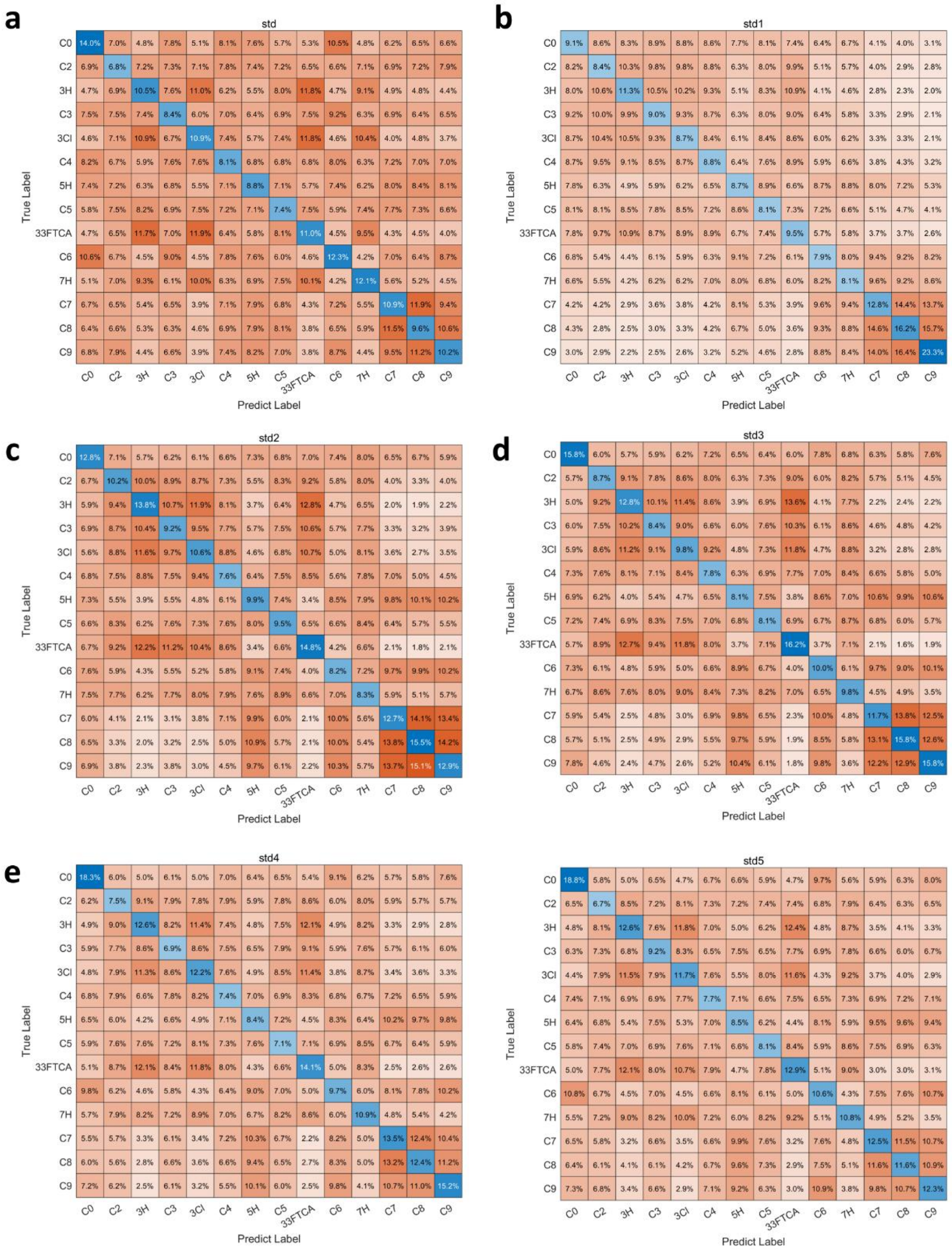

Supplementary Fig. 24. The confusion matrix of 14 analytes in I_σ dimension, (a) original signal, (b) wavelet, (c) 100Hz, (d) 200Hz, (e) 500Hz, (f) 800Hz.

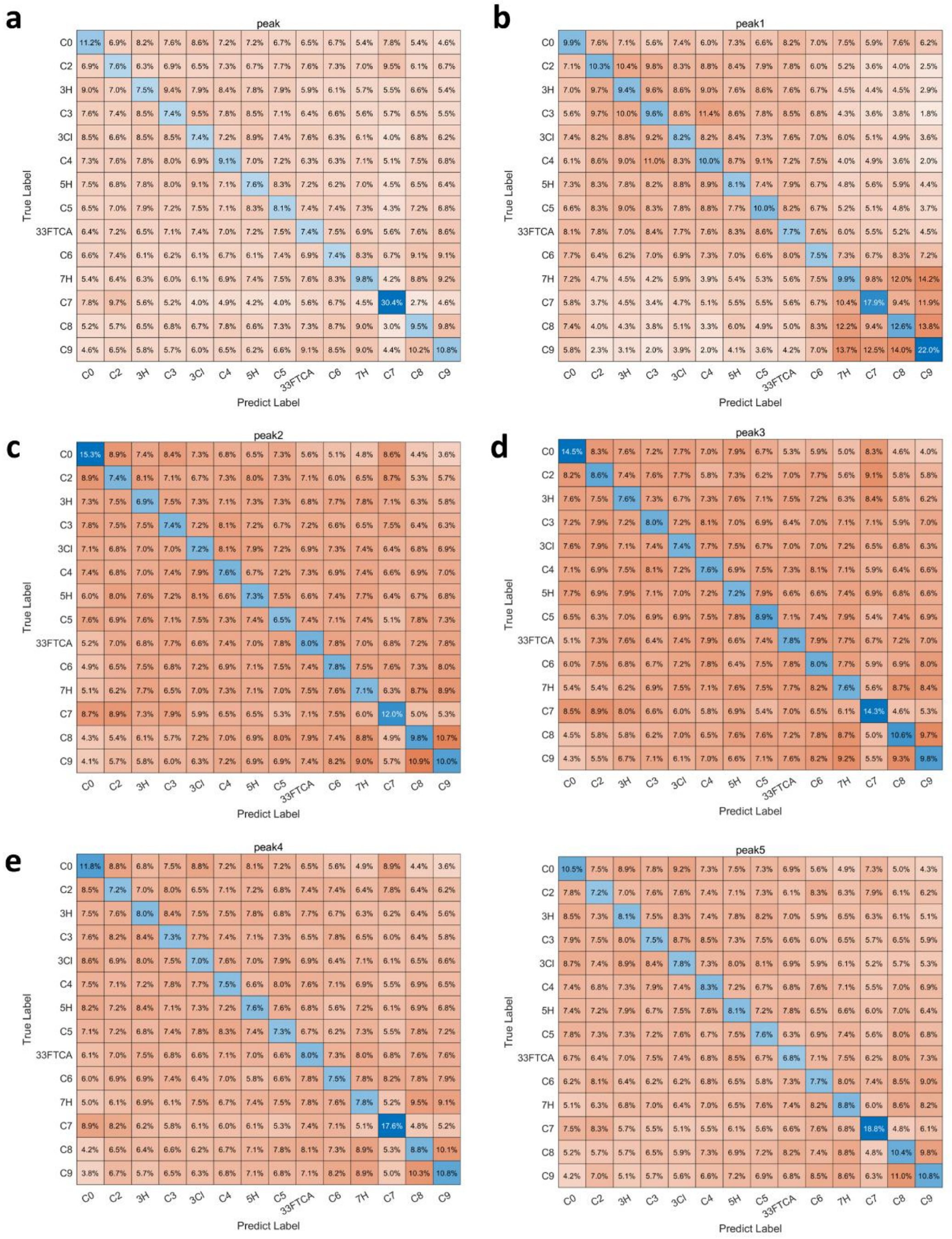

Supplementary Fig. 25. The confusion matrix of 14 analytes in H_{peak} dimension, (a) original signal, (b) wavelet, (c) 100Hz, (d) 200Hz, (e) 500Hz, (f) 800Hz.

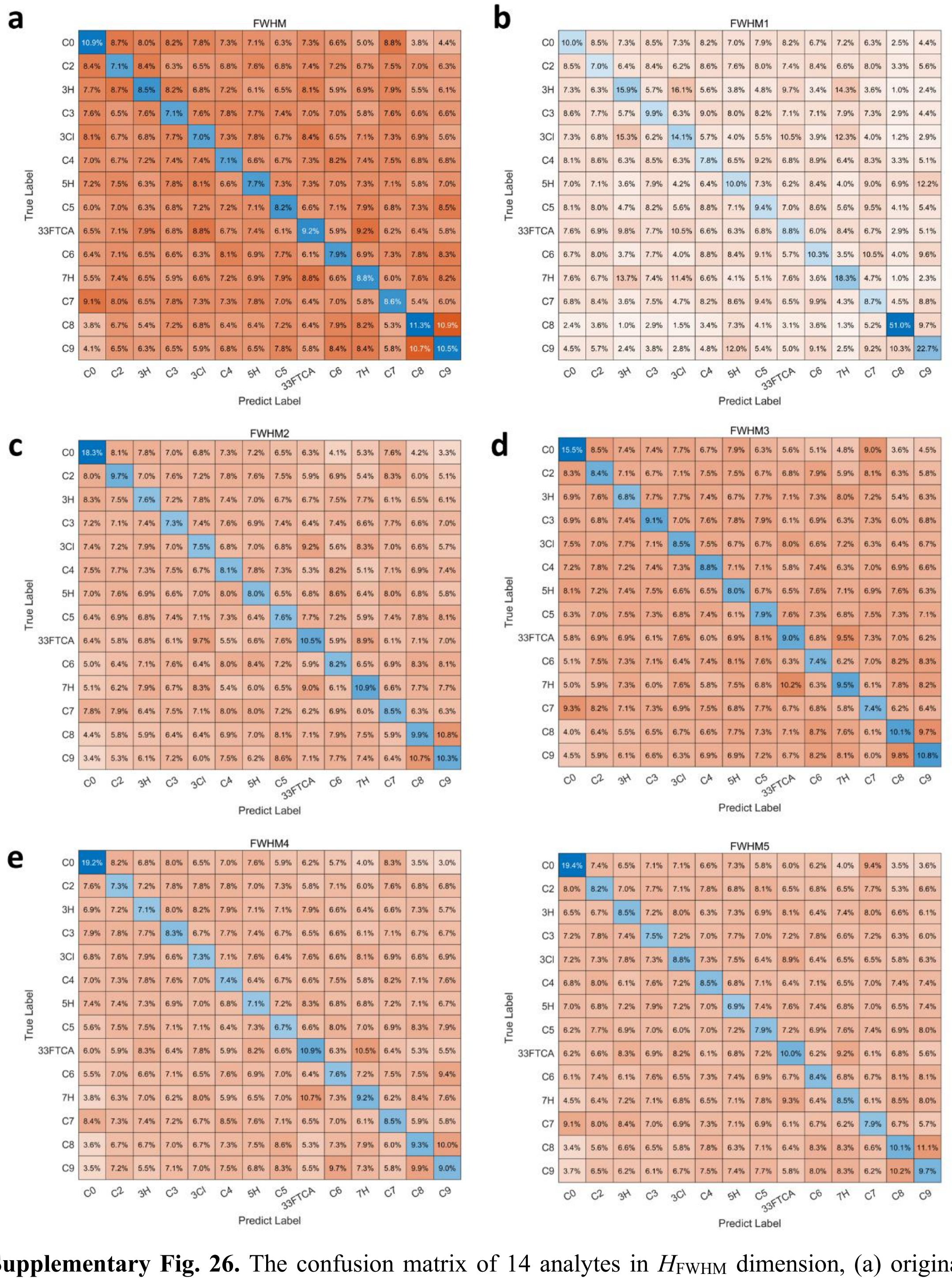

Supplementary Fig. 26. The confusion matrix of 14 analytes in H_{FWHM} dimension, (a) original signal, (b) wavelet, (c) 100Hz, (d) 200Hz, (e) 500Hz, (f) 800Hz.

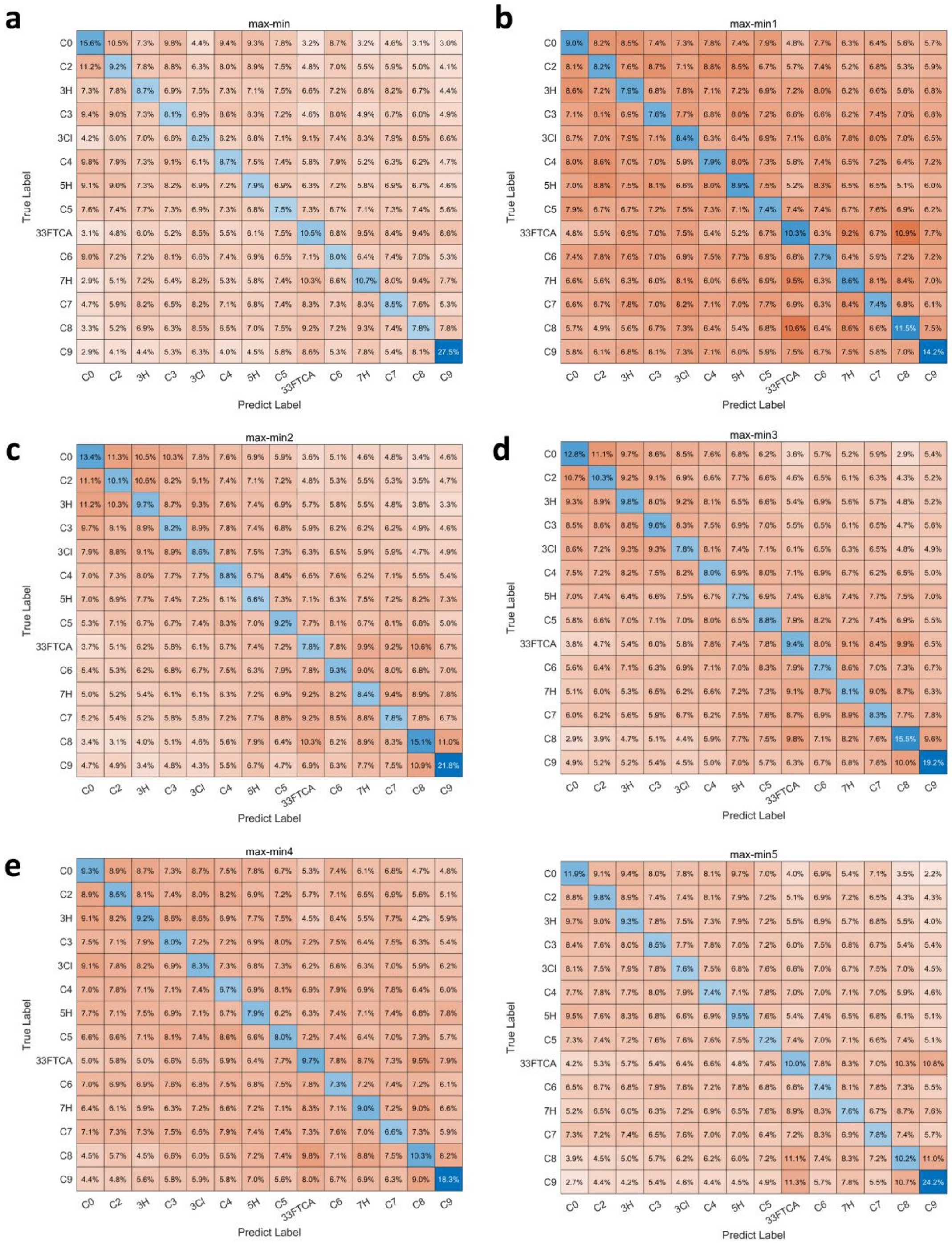

Supplementary Fig. 27. The confusion matrix of 14 analytes in I_{pp} dimension, (a) original signal, (b) wavelet, (c) 100Hz, (d) 200Hz, (e) 500Hz, (f) 800Hz.

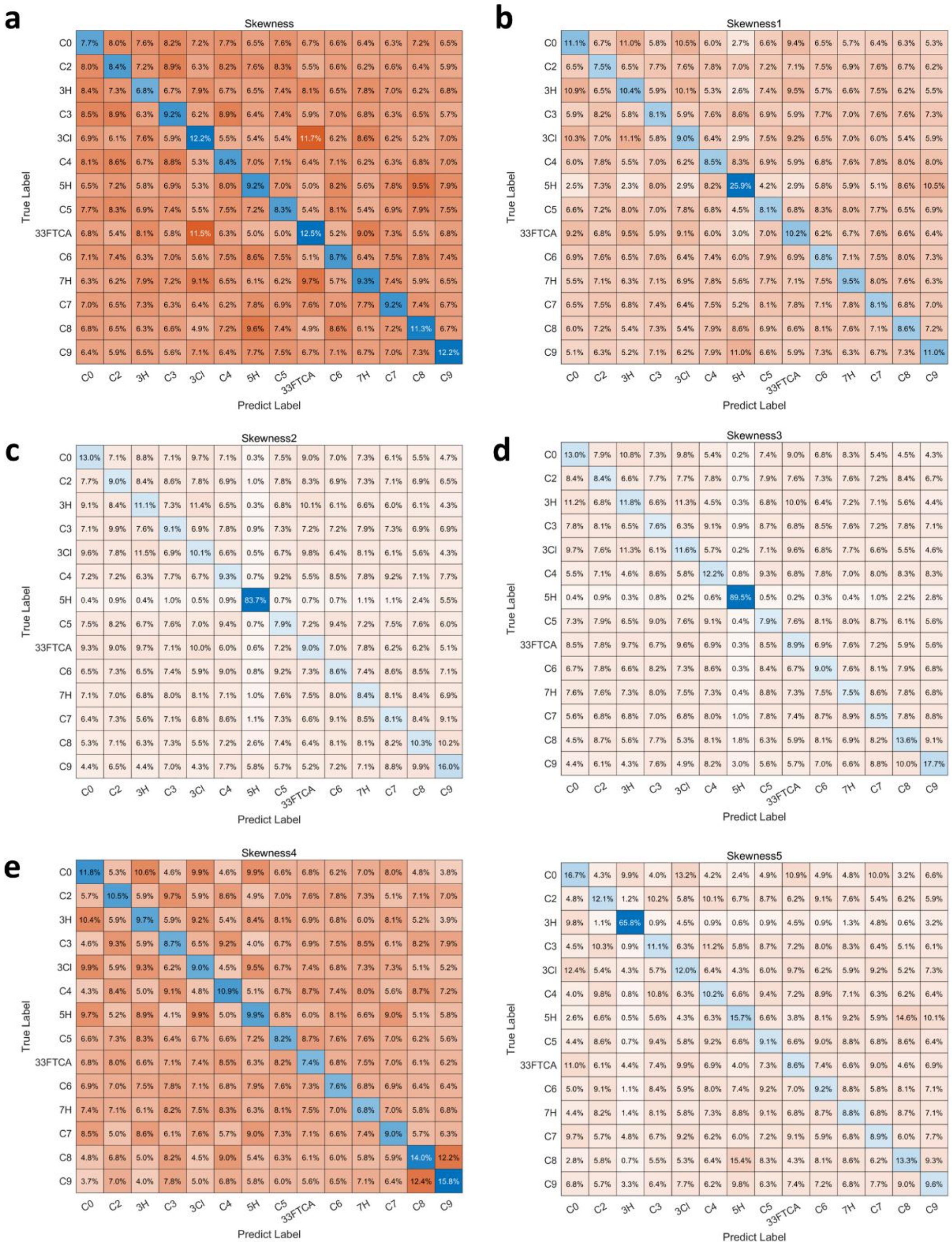

Supplementary Fig. 28. The confusion matrix of 14 analytes in H_{skew} dimension, (a) original signal, (b) wavelet, (c) 100Hz, (d) 200Hz, (e) 500Hz, (f) 800Hz.

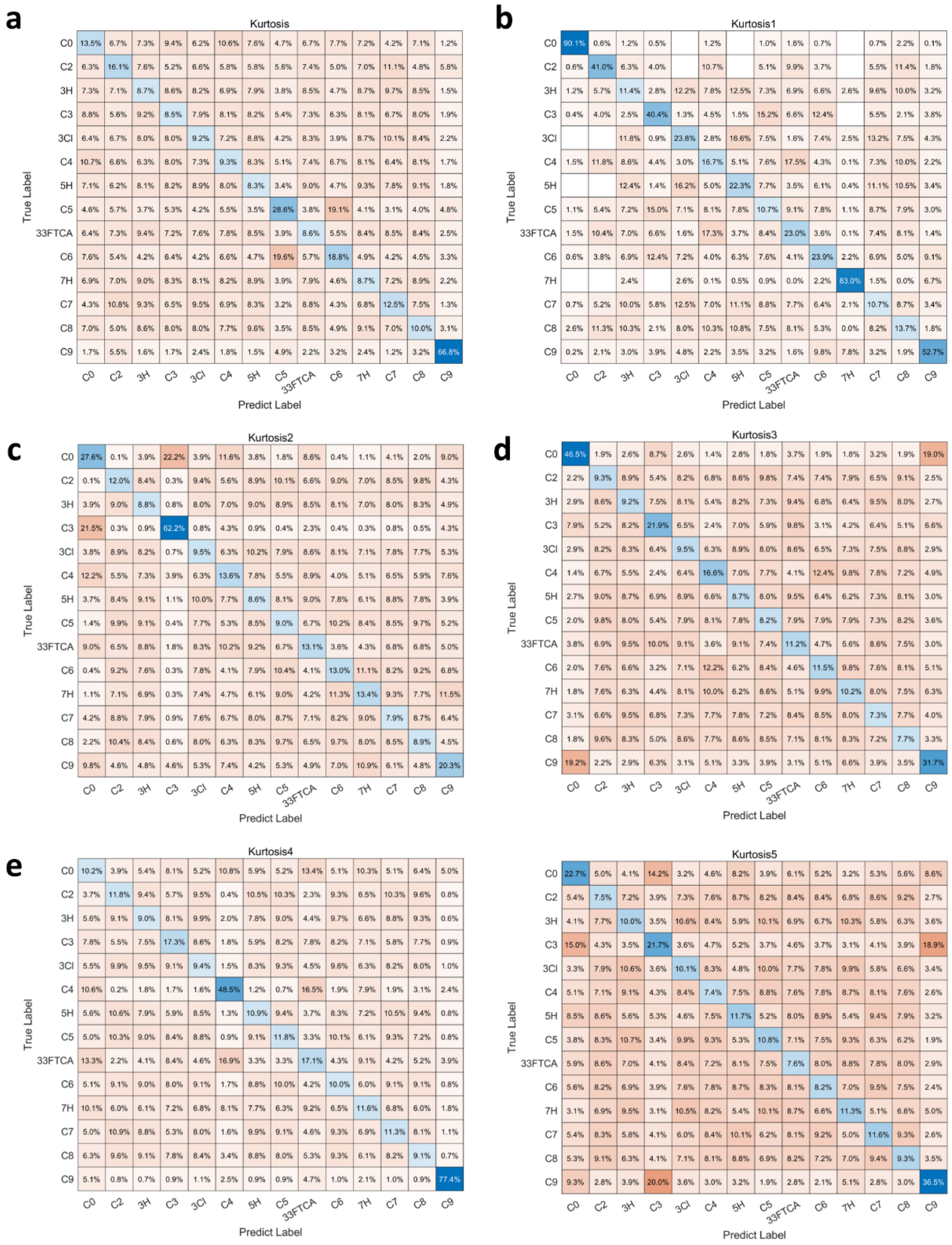

Supplementary Fig. 29. The confusion matrix of 14 analytes in H_{kurt} dimension, (a) original signal, (b) wavelet, (c) 100Hz, (d) 200Hz, (e) 500Hz, (f) 800Hz.

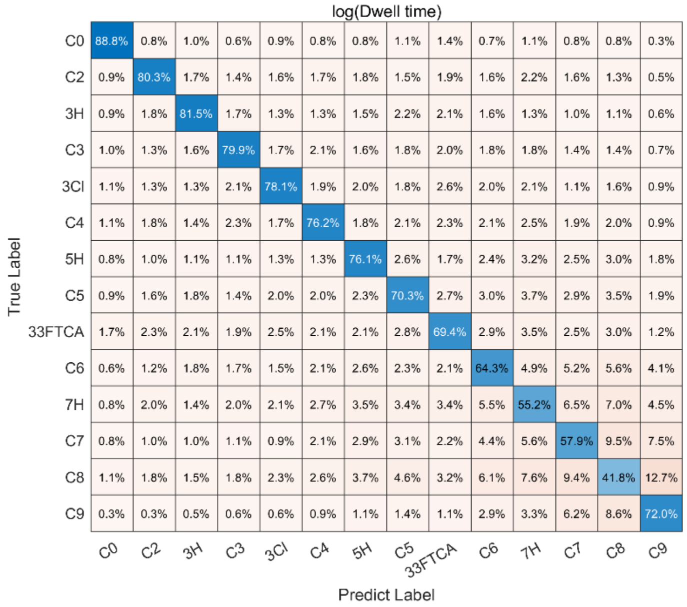

Supplementary Fig. 30. The confusion matrix of 14 analytes in τ_{on} dimension.

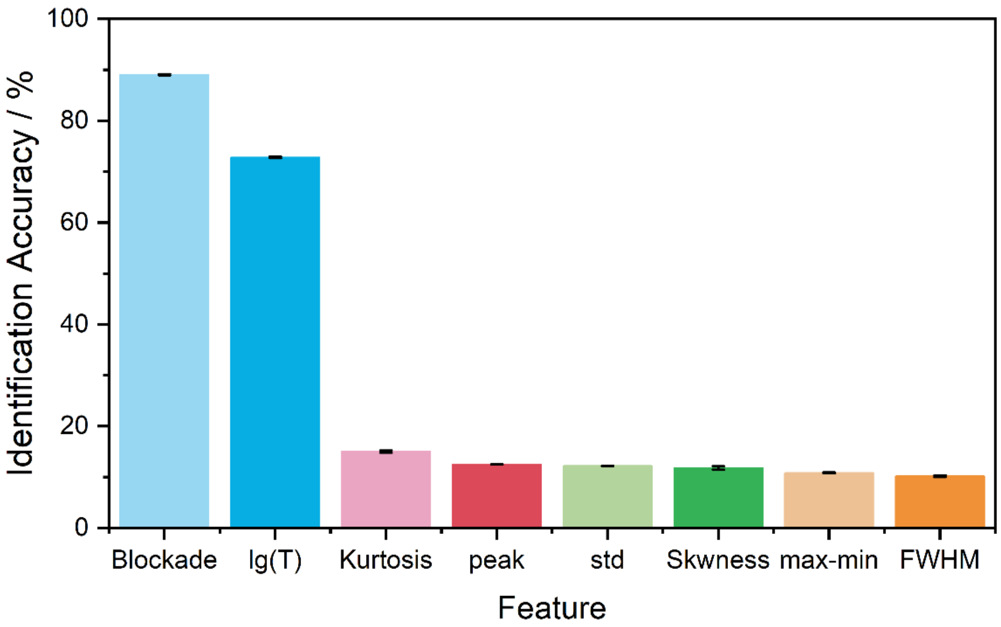

Supplementary Fig. 31. The identification accuracy of 11 analytes in single feature dimension, arranged according to the decrease order of identification accuracy. The error bars were standard deviations calculated from five parallel classifications.

iii Classification performances of different classifiers

Supplementary Table 2. Classification accuracies of all 13 PFCAs and R_6 probe based on different classifiers.

Classifier	Accuracy / %
Fine Tree	99.6
Medium Tree	97.7
Coarse Tree	35.7
Linear Discriminant	99.2
Quadratic Discriminant	99.6
Gaussian Naive Bayes	97.6
Kernel Naive Bayes	98.8
Linear SVM	99.9
Quadratic SVM	99.8
Cubic SVM	99.8
Fine Gaussian SVM	82.3
Medium Gaussian SVM	99.1
Coarse Gaussian SVM	99.5
Fine KNN	95.3
Medium KNN	92.4
Coarse KNN	93.7
Cosine KNN	Training time over 10 minutes
Cubic KNN	95.7
Weighted KNN	95.9
Boosted Trees	99.5
Bagged Trees	99.9
Subspace Discriminant	99.0
Subspace KNN	96.8
RUSBoost Trees	97.6
Narrow Neural Network	99.8
Medium Neural Network	99.8
Wide Neural Network	99.8
Bilayered Neural Network	99.8
Trilayered Neural Network	99.7
SVM Kernel	93.1
Logistic Regression Kernel	96.4

iv Rank of feature importances with different dataset sizes

Supplementary Table 3. The times of features appear in the top 18, 28, 30 in 12 parallel tests, dataset size was 2000. The features without number were extracted from 2000 Hz, and with 1, 2, 3, 4, 5 were extracted from wavelet, lowpass filter at 100, 200, 500 and 800 Hz.

Feature_2000	18	28	30	Mean	Std
Blockade	12	12	12	12.00	0.00
Kurtosis_2	12	12	12	12.00	0.00
Kurtosis_4	12	12	12	12.00	0.00
Kurtosis_5	11	12	12	11.67	0.47
peak	9	12	12	11.00	1.41
Skewness_1	9	11	12	10.67	1.25
std_1	9	10	12	10.33	1.25
peak_1	7	12	12	10.33	2.36
FWHM	9	10	11	10.00	0.82
Kurtosis	7	11	11	9.67	1.89
Kurtosis_3	6	11	11	9.33	2.36
max-min	6	11	11	9.33	2.36
peak_3	5	11	11	9.00	2.83
FWHM_2	6	10	11	9.00	2.16
Skewness_3	6	10	10	8.67	1.89
std	2	12	12	8.67	4.71
Kurtosis_1	3	11	12	8.67	4.03
peak_2	7	9	9	8.33	0.94
log(Dwell time)	7	8	10	8.33	1.25
max-min_1	6	9	10	8.33	1.70
Skewness_2	6	9	10	8.33	1.70
FWHM_3	5	9	10	8.00	2.16
FWHM_1	4	8	9	7.00	2.16
std_2	5	7	9	7.00	1.63
Skewness	3	8	9	6.67	2.62
Skewness_4	6	7	7	6.67	0.47
peak_4	3	8	9	6.67	2.62
max-min_4	3	7	9	6.33	2.49
max-min_2	3	8	8	6.33	2.36
max-min_3	4	6	8	6.00	1.63
FWHM_4	4	7	7	6.00	1.41
FWHM_5	4	6	7	5.67	1.25
Skewness_5	1	7	7	5.00	2.83
std_3	2	5	6	4.33	1.70
peak_5	3	3	6	4.00	1.41
std_4	2	5	5	4.00	1.41
std_5	2	3	4	3.00	0.82
max-min_5	2	2	3	2.33	0.47
Blockade_1	1	1	1	1.00	0.00
Blockade_3	1	1	1	1.00	0.00
Blockade_2					
Blockade_4					
Blockade_5					

Supplementary Table 4. The times of features appear in the top 18, 25, 30 in 20 parallel tests, dataset size was 200. The features without number were extracted from 2000 Hz, and with 1, 2, 3, 4, 5 were extracted from wavelet, lowpass filter at 100, 200, 500 and 800 Hz.

Feature_200	18	25	30	Mean	Std
Blockade	20	20	20	20.00	0.00
Kurtosis_4	20	20	20	20.00	0.00
Kurtosis_2	18	20	20	19.33	0.94
std	16	19	19	18.00	1.41
Skewness	16	18	18	17.33	0.94
Kurtosis_3	12	19	19	16.67	3.30
Kurtosis	15	16	18	16.33	1.25
log(Dwell time)	13	17	18	16.00	2.16
peak_1	12	17	19	16.00	2.94
FWHM	11	17	19	15.67	3.40
Kurtosis_1	13	15	17	15.00	1.63
peak	10	15	20	15.00	4.08
max-min_1	11	16	18	15.00	2.94
Skewness_1	12	16	17	15.00	2.16
max-min	9	16	19	14.67	4.19
FWHM_1	9	16	19	14.67	4.19
FWHM_3	11	15	17	14.33	2.49
Skewness_2	11	14	16	13.67	2.05
std_3	7	15	17	13.00	4.32
peak_2	10	12	17	13.00	2.94
std_1	7	14	17	12.67	4.19
FWHM_2	7	11	17	11.67	4.11
max-min_2	7	11	14	10.67	2.87
Skewness_3	7	9	14	10.00	2.94
std_2	6	11	13	10.00	2.94
max-min_4	6	10	11	9.00	2.16
peak_3	4	11	12	9.00	3.56
std_5	8	8	11	9.00	1.41
FWHM_5	5	9	11	8.33	2.49
Skewness_4	5	7	12	8.00	2.94
std_4	4	7	11	7.33	2.87
peak_4	4	7	11	7.33	2.87
Kurtosis_5	3	8	10	7.00	2.94
max-min_5	5	7	9	7.00	1.63
FWHM_4	5	6	9	6.67	1.70
max-min_3	3	5	11	6.33	3.40
Skewness_5	3	5	10	6.00	2.94
Blockade_2	4	6	8	6.00	1.63
peak_5	3	6	6	5.00	1.41
Blockade_1	2	3	9	4.67	3.09
Blockade_3	4	4	4	4.00	0.00
Blockade_4	2	2	3	2.33	0.47
Blockade_5					

Supplementary Table 5. The times of features appear in the top 18, 21, 23 in 35 parallel tests, dataset size was 20.

The features without number were extracted from 2000 Hz, and with 1, 2, 3, 4, 5 were extracted from wavelet, lowpass filter at 100, 200, 500 and 800 Hz.

**Feature_20**	**18**	**21**	**23**	**Mean**	**Std**
Blockade	35	35	35	35.00	0.00
max-min	30	34	34	32.67	1.89
log(Dwell time)	31	33	34	32.67	1.25
peak	30	32	33	31.67	1.25
Skewness	29	31	33	31.00	1.63
Kurtosis	28	31	31	30.00	1.41
FWHM	27	29	32	29.33	2.05
Std	28	28	28	28.00	0.00
peak_1	25	28	30	27.67	2.05
FWHM_1	26	28	29	27.67	1.25
Skewness_1	25	29	29	27.67	1.89
Blockade_1	26	28	28	27.33	0.94
std_1	25	27	27	26.33	0.94
Kurtosis_1	21	25	26	24.00	2.16
max-min_1	20	24	27	23.67	2.87
peak_2	16	24	29	23.00	5.35
FWHM_2	14	22	25	20.33	4.64
std_2	15	21	24	20.00	3.74
Kurtosis_2	14	19	21	18.00	2.94
Kurtosis_4	16	16	18	16.67	0.94
max-min_2	13	14	23	16.67	4.50
peak_3	14	17	17	16.00	1.41
Skewness_2	10	15	20	15.00	4.08
std_3	9	13	19	13.67	4.11
Kurtosis_3	11	12	13	12.00	0.82
Blockade_2	9	13	14	12.00	2.16
Skewness_3	9	10	11	10.00	0.82
max-min_3	6	11	13	10.00	2.94
peak_4	9	10	10	9.67	0.47
Blockade_3	4	9	13	8.67	3.68
Blockade_4	8	8	8	8.00	0.00
FWHM_3	6	8	9	7.67	1.25
max-min_4	5	8	9	7.33	1.70
std_5	6	6	8	6.67	0.94
FWHM_4	4	7	9	6.67	2.05
Skewness_4	5	5	7	5.67	0.94
peak_5	4	5	6	5.00	0.82
max-min_5	4	5	5	4.67	0.47
Blockade_5	4	4	6	4.67	0.94
std_4	3	5	5	4.33	0.94
Kurtosis_5	2	2	3	2.33	0.47
FWHM_5	2	2	2	2.00	0.00
Skewness_5	1	1	2	1.33	0.47

v Clustering to differentiate PFCA and FA of same blockade

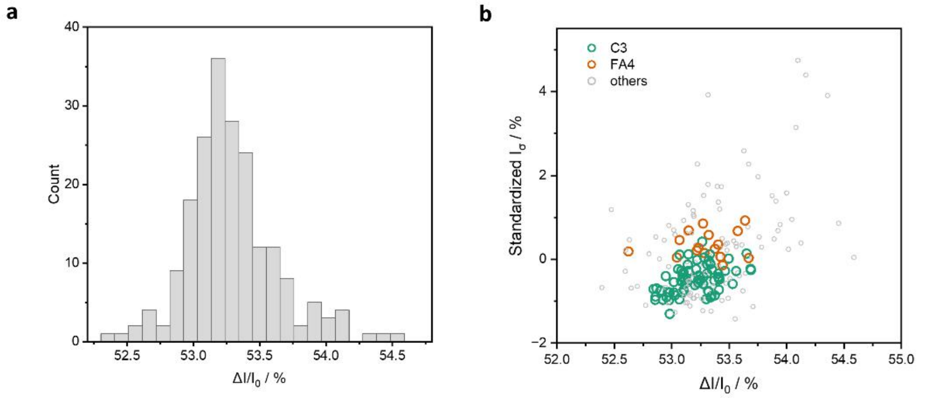

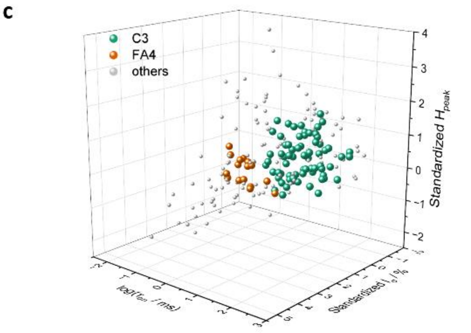

d

Features	C3	FA4
Volume / A^3	2762.08	2762.82
$\Delta I/I_0$ / %	53.20	53.29
I_σ / %	1.46	1.83
log(T_{on} / ms)	1.06	0.59
H_{peak}	0.29	0.25
Concentration Ratio	4.00	1.00
Signal number Ratio	3.89	1.00

Supplementary Fig. 32. (a) One-, (b) two- and (c) three-dimensional distribution of cluster results of C3 and butyric acid (FA4), whose current blockades are extremely close. (d) Mean values of molecular volumes, $\Delta I/I_0$, I_σ, τ_{on} and H_{peak} of C3 and FA4. The concentration ratio of C3 and FA4 in mixed samples was set as 4:1, and the signal number ratio achieved by unsupervised clustering was 3.89:1.

IV Standard-free quantification

i Capture rates for linear PFCAs of different chain lengths

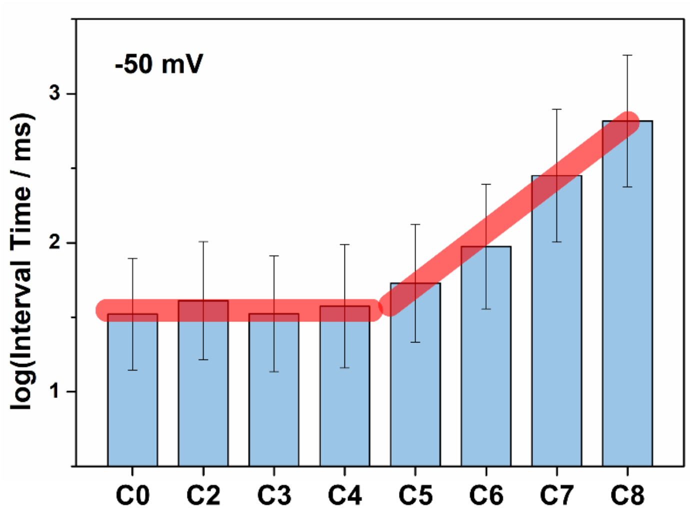

Supplementary Fig. 33. Comparison of the interval time for C0 and C2 to C8 at -50 mV. The error bars were standard deviations obtained from the histograms of interval times of C0 and C2 to C8 at -50 mV.

ii Standard-free quantification of short-chain PFCAs

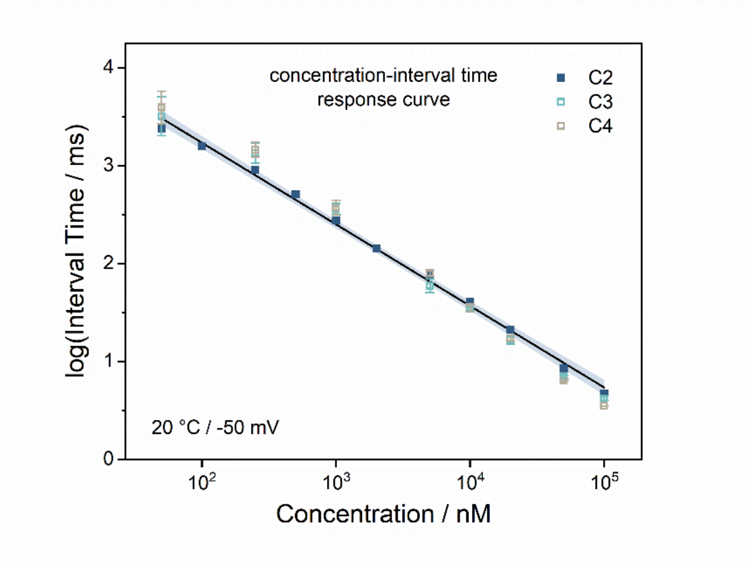

Supplementary Fig. 34. Shared concentration-interval time response curve of C2, C3 and C4 (linear fit, $R^2 = 9961$). The error bars were standard deviations calculated from at least three parallel measurements.

iii Simultaneous quantification ability in mixed analytes

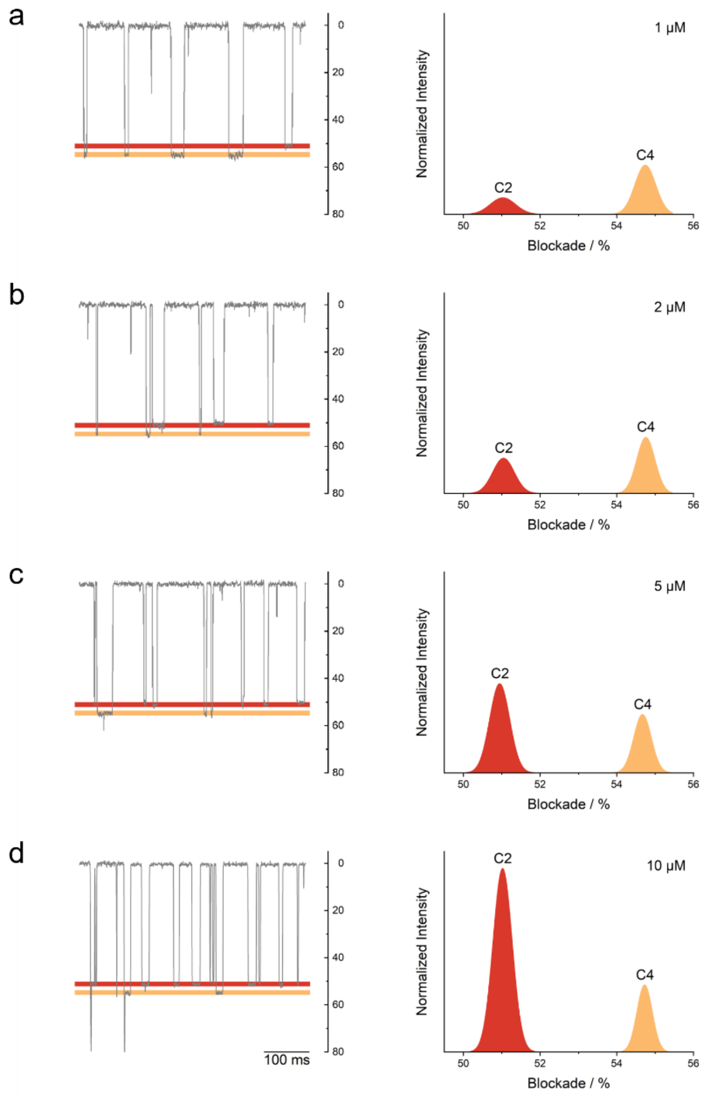

Supplementary Fig. 35. (a-d) Representative current traces (left) and signal intensity histograms (right) of C2-R_6 with different concentration (1~10 μM) under the interference of C4-R_6.

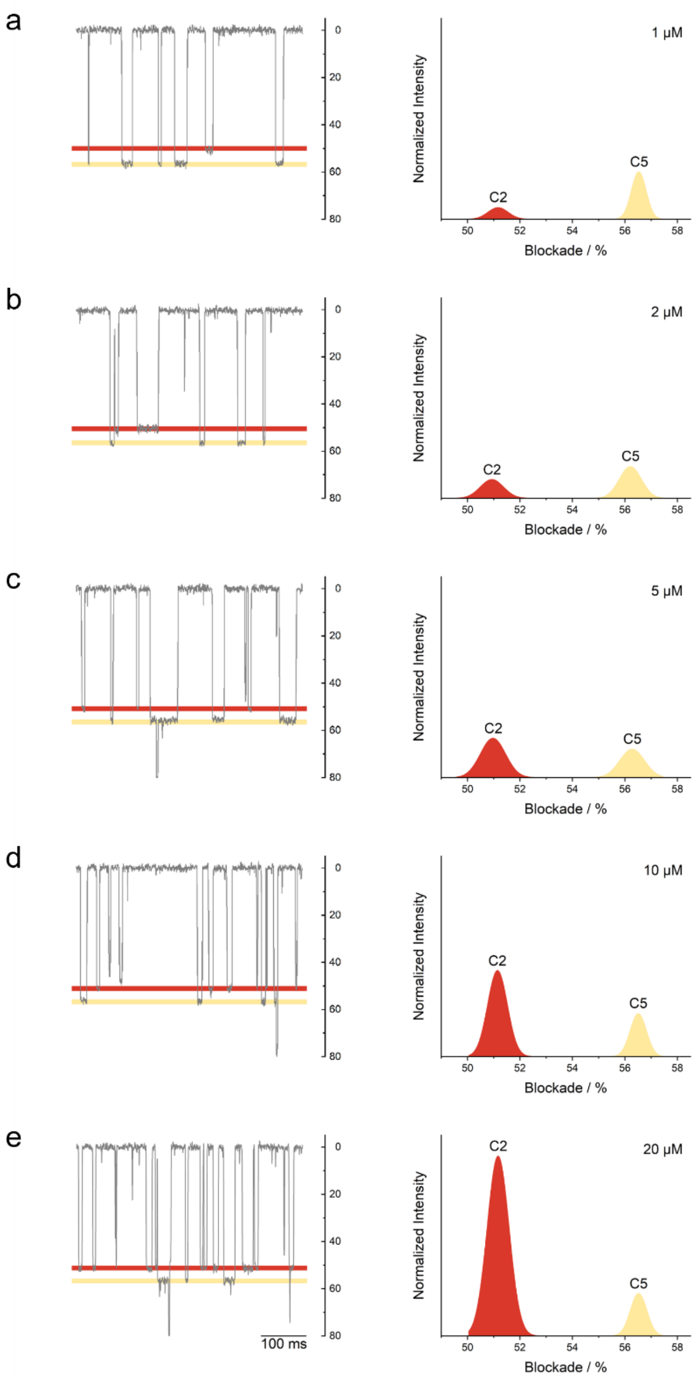

Supplementary Fig. 36. (a-e) Representative current traces (left) and signal intensity histograms (right) of C2-R_6 with different concentration (1~20 μM) under the interference of C5-R_6.

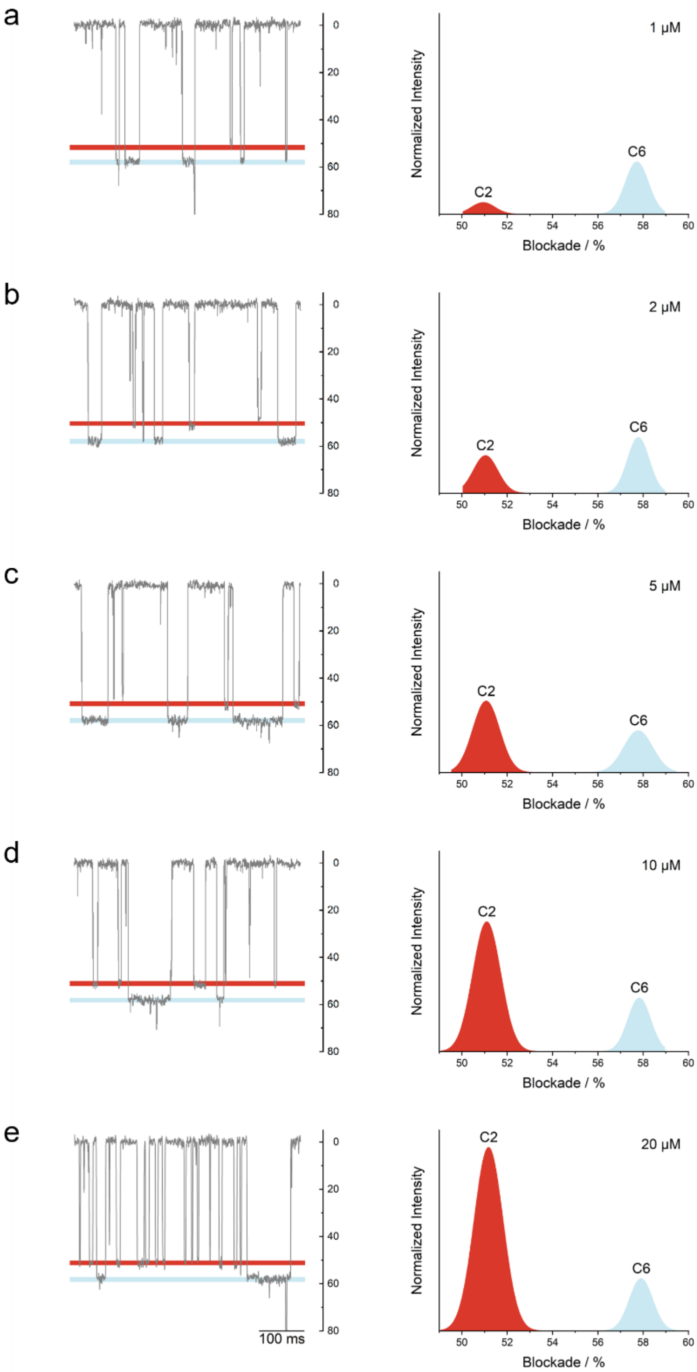

Supplementary Fig. 37. (a-e) Representative current traces (left) and signal intensity histograms (right) of C2-R_6 with different concentration (1~20 μM) under the interference of C6-R_6.

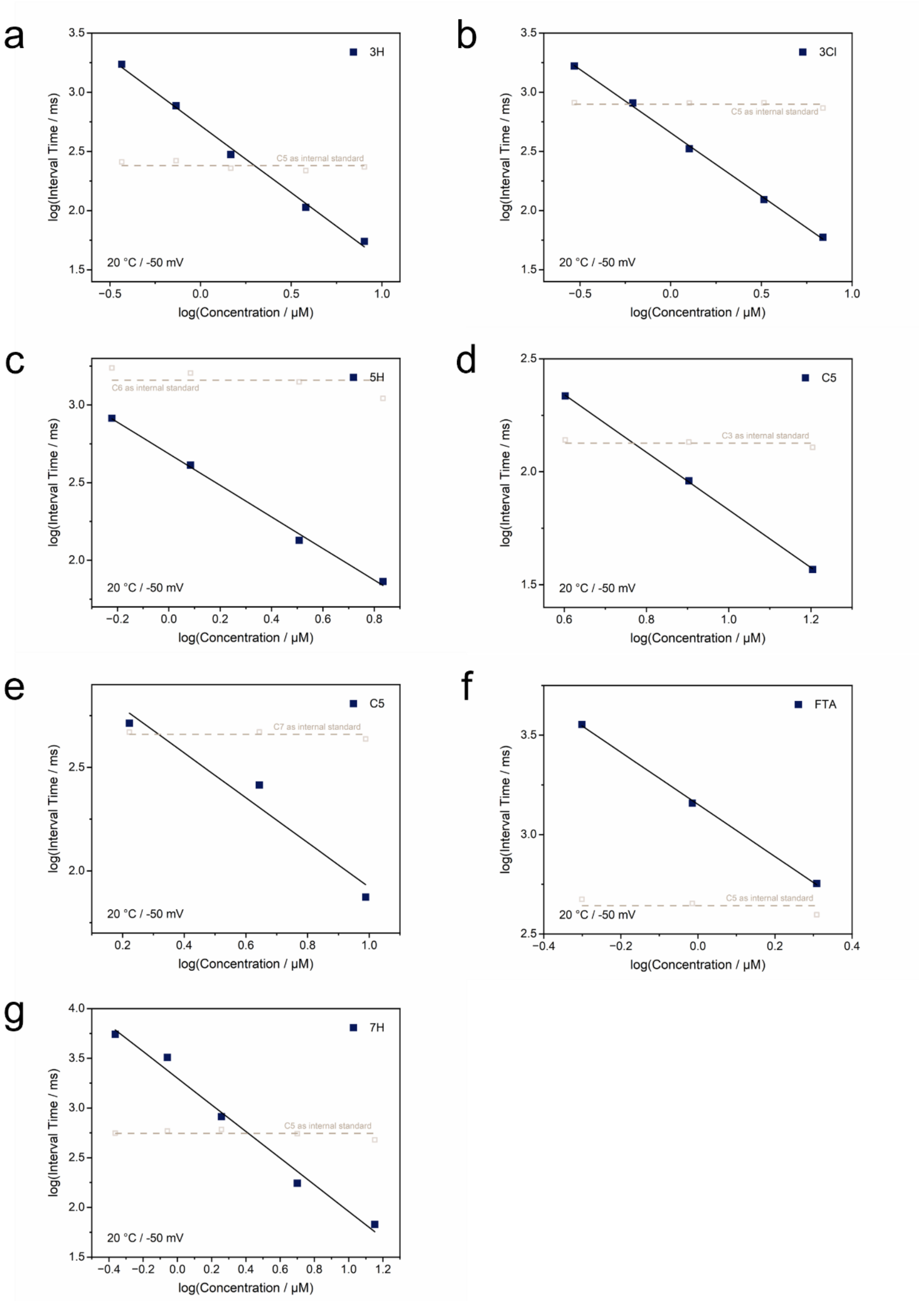

Supplementary Fig. 38. Simultaneous quantification instances of (a) 3H with C5-R_6 interference, (b) 3Cl with C5-R_6 interference, (c) 5H with C6-R_6 interference, (d) C5 with C3-R_6 interference, (e) C5 with C7-R_6 interference, (f) FTA with C5-R_6 interference, and (g) 7H with C5-R_6 interference (linear fit, R^2 = 0.9509-0.9999).

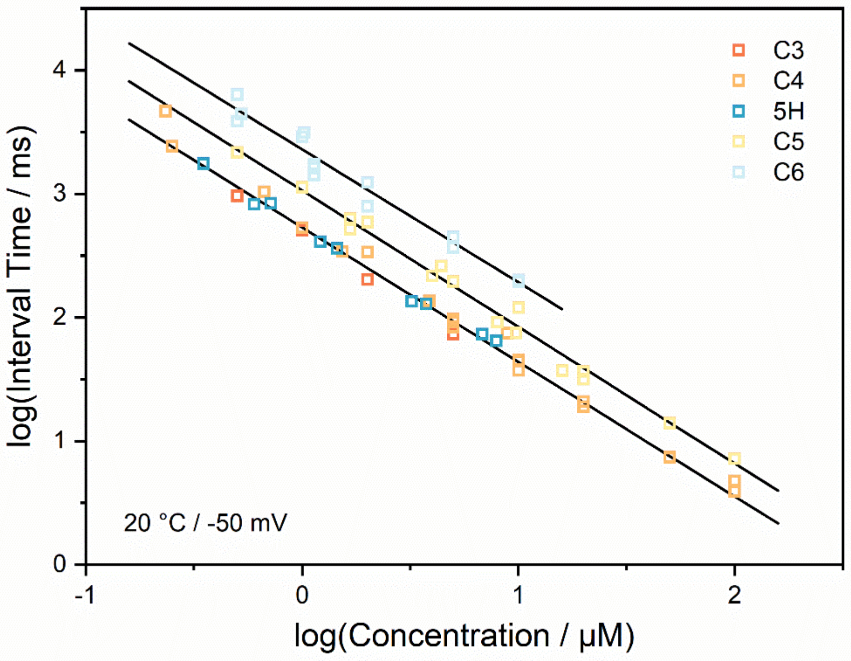

Supplementary Fig. 39. Calibration curves of C3, C4, 5H, C5 and C6 measured in the presence of internal standards of fixed concentrations (linear fit, R^2 = 0.9632-0.9909).

iv Robust quantification ability under various interferences

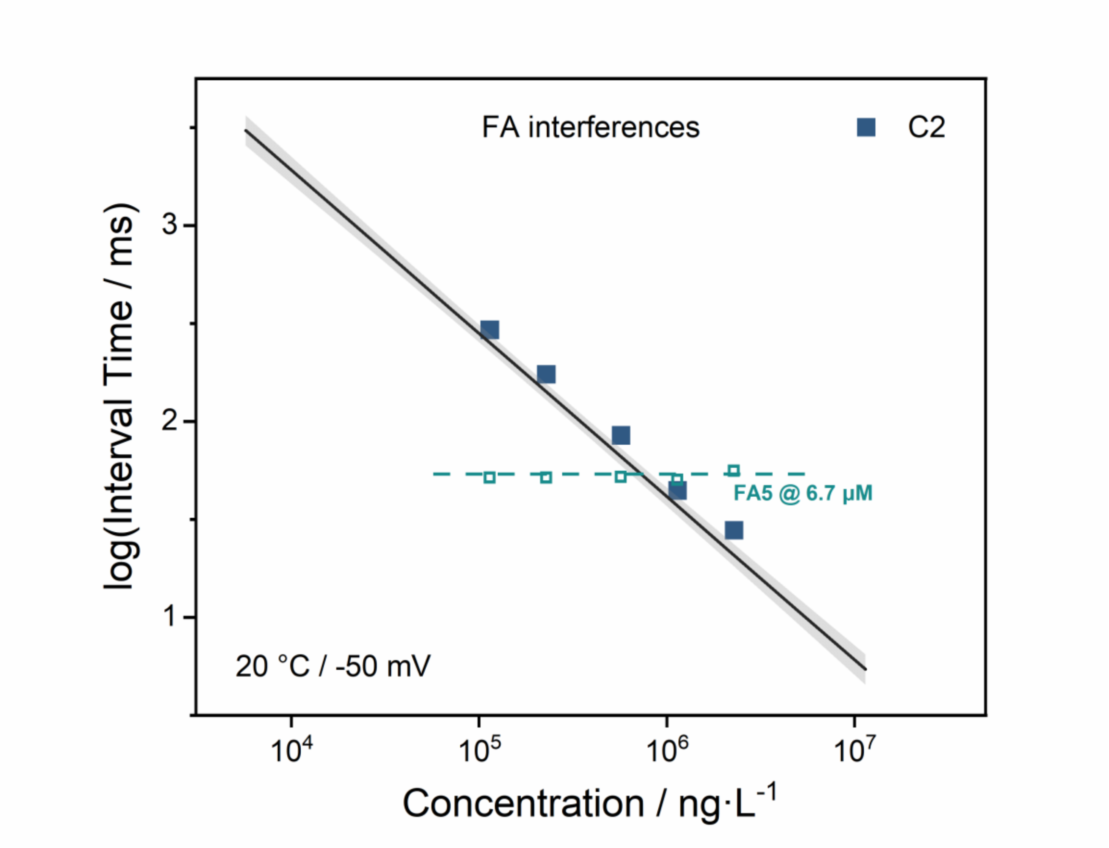

Supplementary Fig. 40. Quantification of C2-R_6 under tethered interference of valeric acid (FA5-R_6) (linear fit, R^2 = 0.9961).

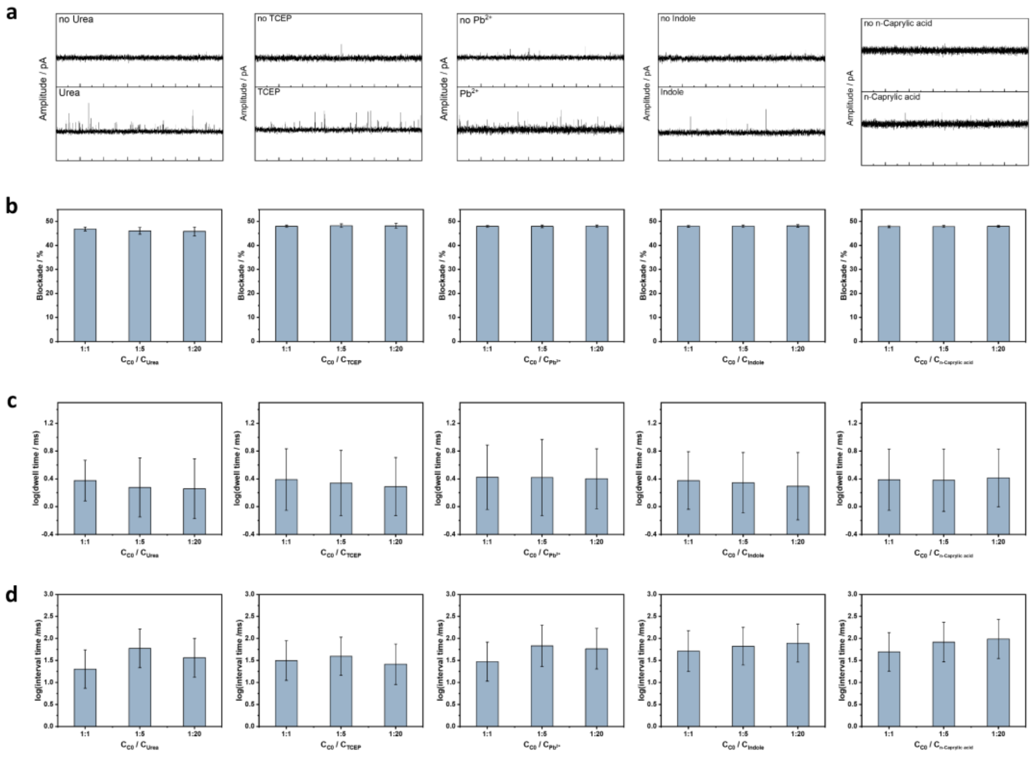

Supplementary Fig. 41. (a) Raw current traces without and with untethered interference - urea, Tris(2-carboxyethyl)phosphine (TCEP), Pb^{2+}, indole, caprylic acid. (b-d) $\Delta I/I_0$, τ_{on} and τ_{off} of C0 at different concentrations of environmental or biological interferences. The concentration of C0 was 4 μM in urea and TCEP, and 2 μM in Pb^{2+}, indole and caprylic acid. The error bars were standard deviations obtained from the histograms of features.

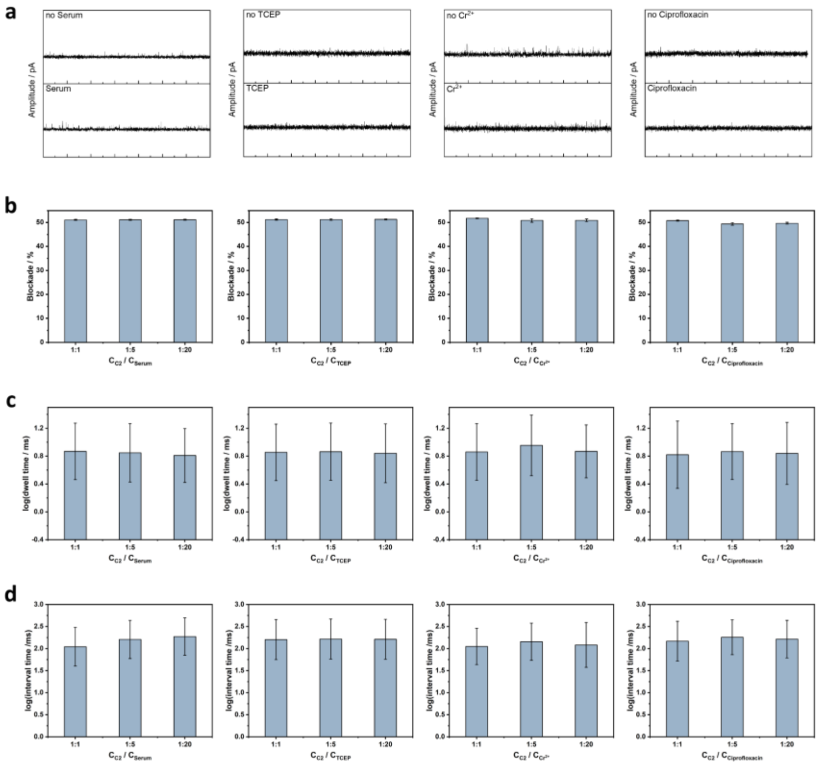

Supplementary Fig. 42. (a) Raw current traces without and with untethered interference - serum, TCEP, Cr^{2+}, Ciprofloxacin. (b-d) $\Delta I/I_0$, τ_{on} and τ_{off} of C2 at different concentrations of environmental or biological interferences. The concentration of C2 was 2 μM. The error bars were standard deviations obtained from the histograms of features.

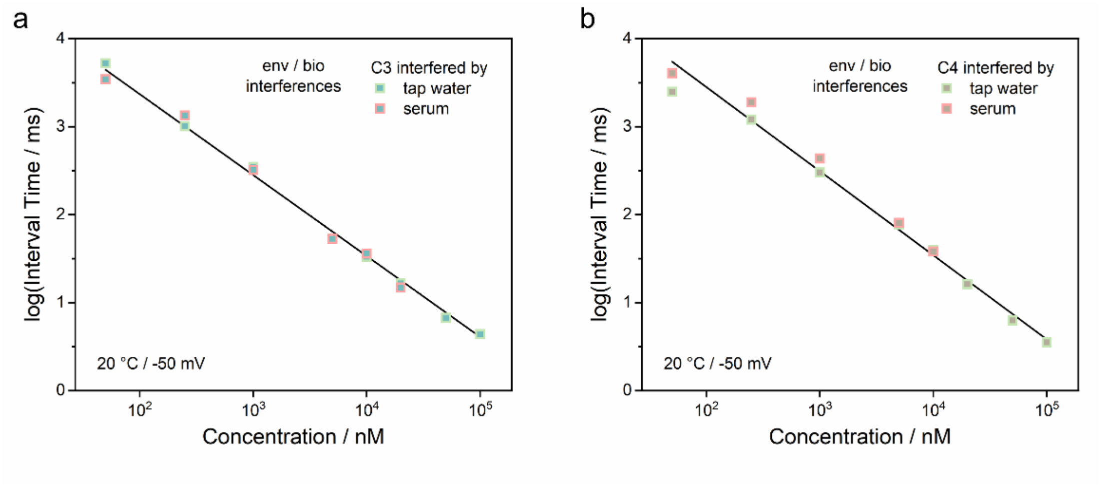

Supplementary Fig. 43. Interference-free quantification of (a) C3 and (b) C4 in presence of tap water or serum at 20°C and -50 mV (linear fit, R^2 = 0.9934-0.9945).

v Strong quantification ability with low limit of detection

Supplementary Table 6. The limit of detection of C2 in previous research.

Year	Journal	LOD	Methods	Ref
2024	*Environ. Sci. Technol*	13 / 110	GC-ECD	3
2024	*Environ. Sci. Technol*	500	LC-MS/MS	4
2023	*Environ. Sci. Technol*	27	IC-MS/MS	5
2023	*Environ. Sci. Technol*	35	HPLC-MS/MS	6
2023	*Environ. Sci. Technol*	172	HPLC-MS/MS	7
2023	*Anal. Chem.*	10	UPLC-MS/MS	8
2022	*Environ. Sci. Technol*	19.5 / 95.1	GC-ECD	9

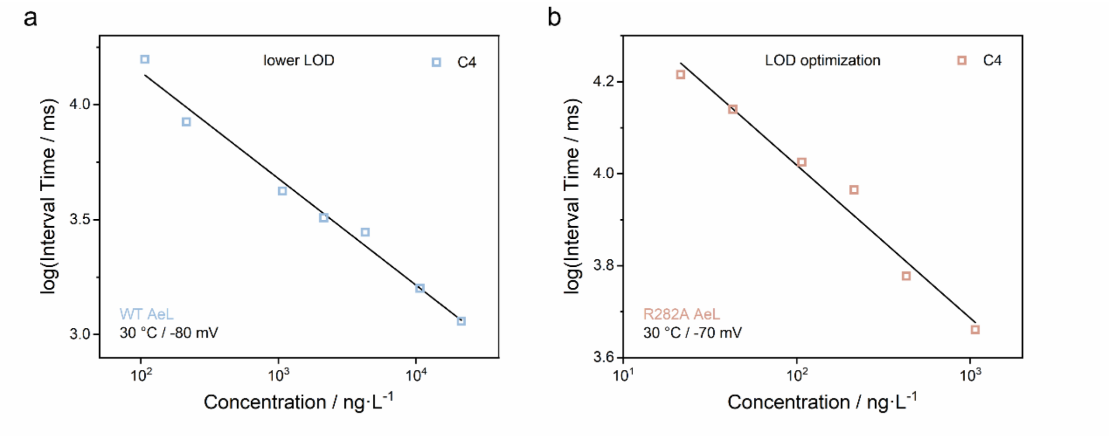

Supplementary Fig. 44. (a) Quantification of C4 by WT AeL at 30°C and -80 mV to lower down the limit of detection (linear fit, $R^2 = 0.9848$). The LOD was 0.5 nM or 107 $ng \cdot L^{-1}$. (b) Quantification of C4 by R282A AeL at 30°C and -70 mV to further optimize the limit of detection (linear fit, $R^2 = 0.9763$). The LOD was 0.1 nM or 21 $ng \cdot L^{-1}$.

V Optimization and applications

i Consistent identification performance under various quantification conditions

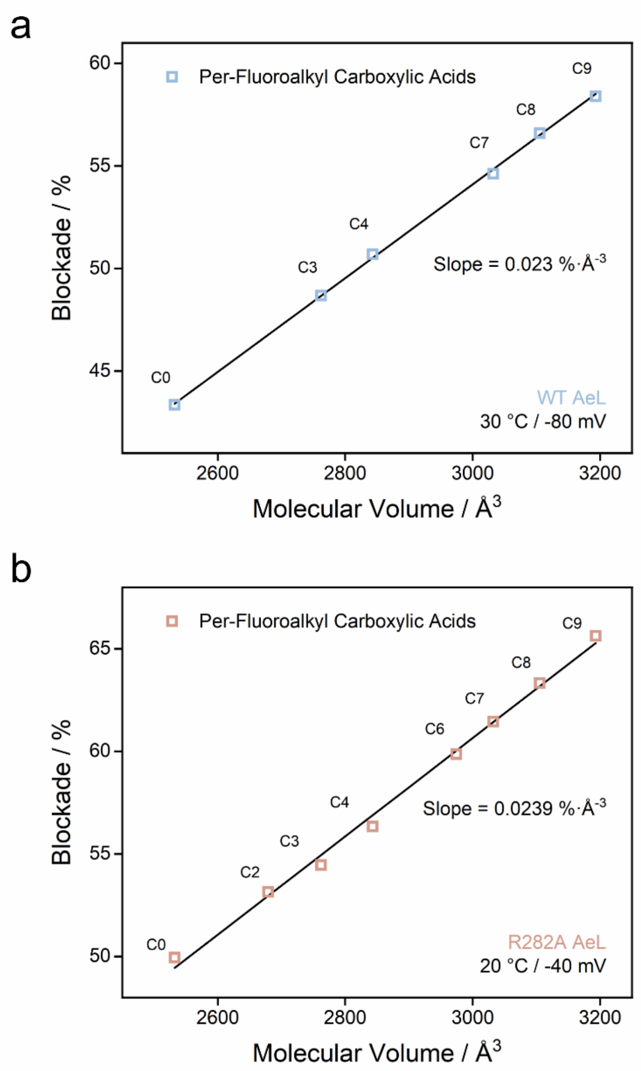

Supplementary Fig. 45. The established linear relationship between the hydrodynamic volume of C0-C9 PFCA-R_6 and the magnitude of their current blockade (a) by WT AeL at 30°C and -80 mV (linear fit, R^2 = 0.9995), and (b) by R282A AeL at 20°C and -40 mV (linear fit, R^2 = 0.9951).

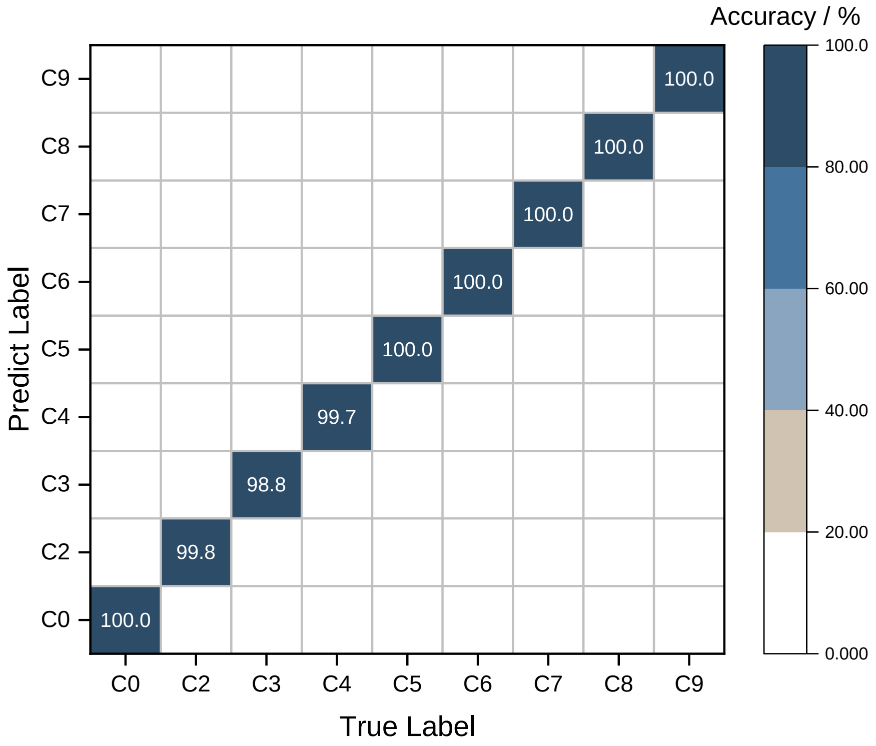

Supplementary Fig. 46. The confusion matrix for identification of 8 PFCAs and R_6 probe by R282A AeL, at 30°C and -70 mV. Identification accuracy was 99.81%.

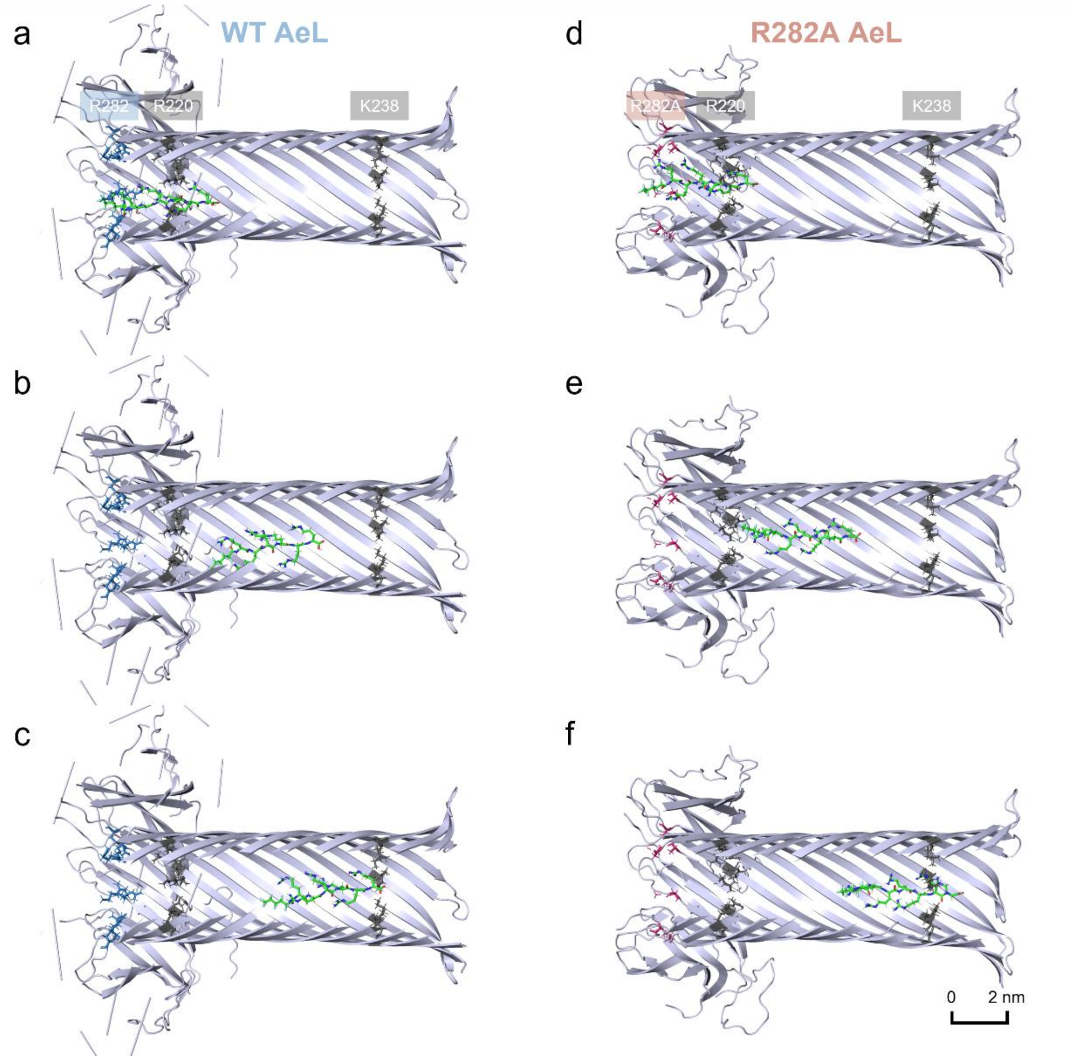

Supplementary Fig. 47. Visualized entry, dwell and exit events (a-c, d-f) of C6-R_6 conjugate in WT/R282A AeL nanopores.

ii Energy barrier regulation towards standard-free quantification

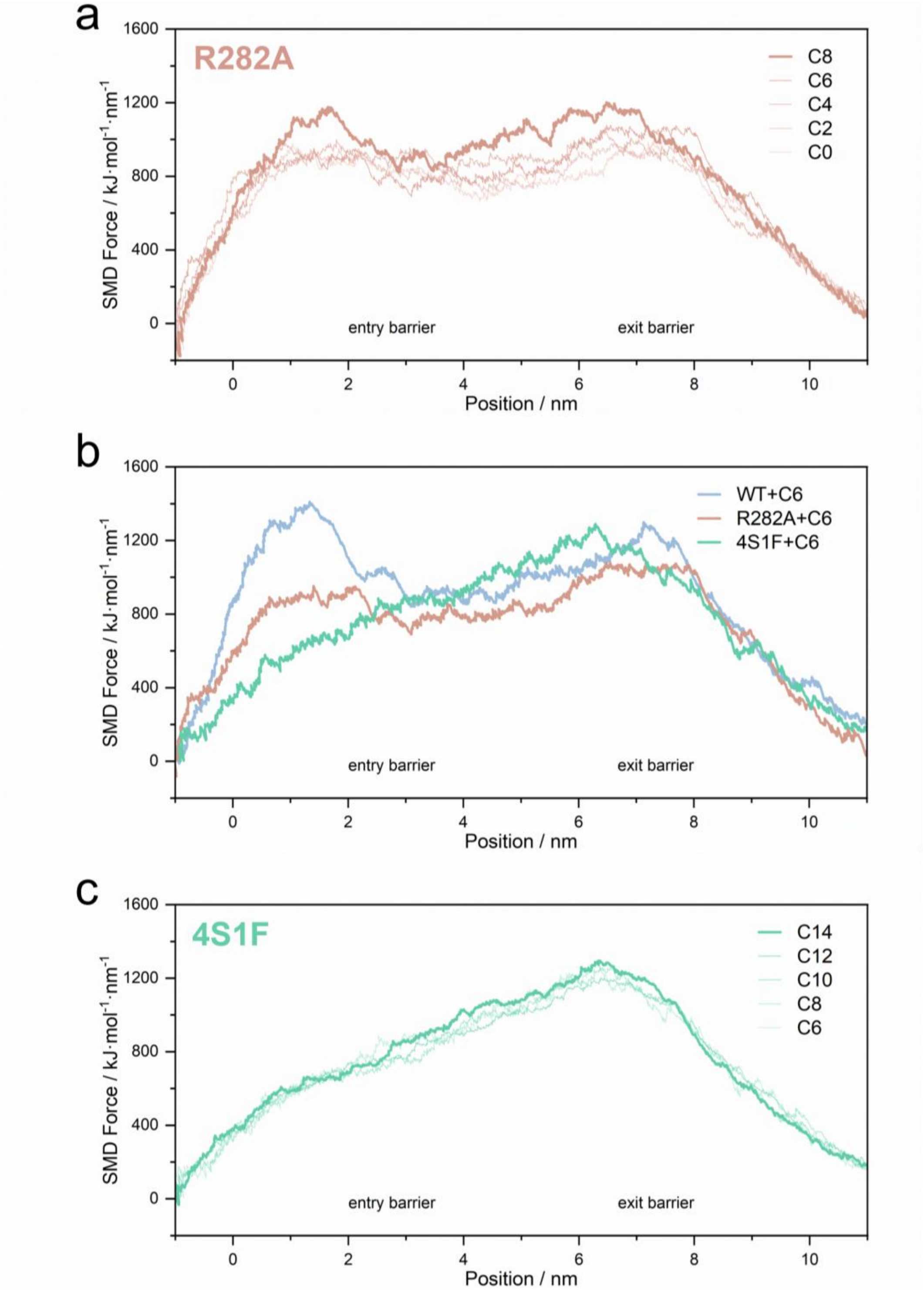

Supplementary Fig. 48. (a) Required force to pull C0/C2/C4/C6/C8-R_6 conjugate through R282A aerolysin nanopore based on steered molecular dynamics (SMD). (b) Required force to pull C6-R_6 conjugate through WT, R282A, or R282S/D216S/R220S/D222S/A260F (4S1F) aerolysin nanopore based on SMD. (c) Required force to pull C6/C8/C10/C12/C14-R_6 conjugate through 4S1F aerolysin nanopore based on SMD.

iii Blockade prediction and identification performance of PFCA isomers

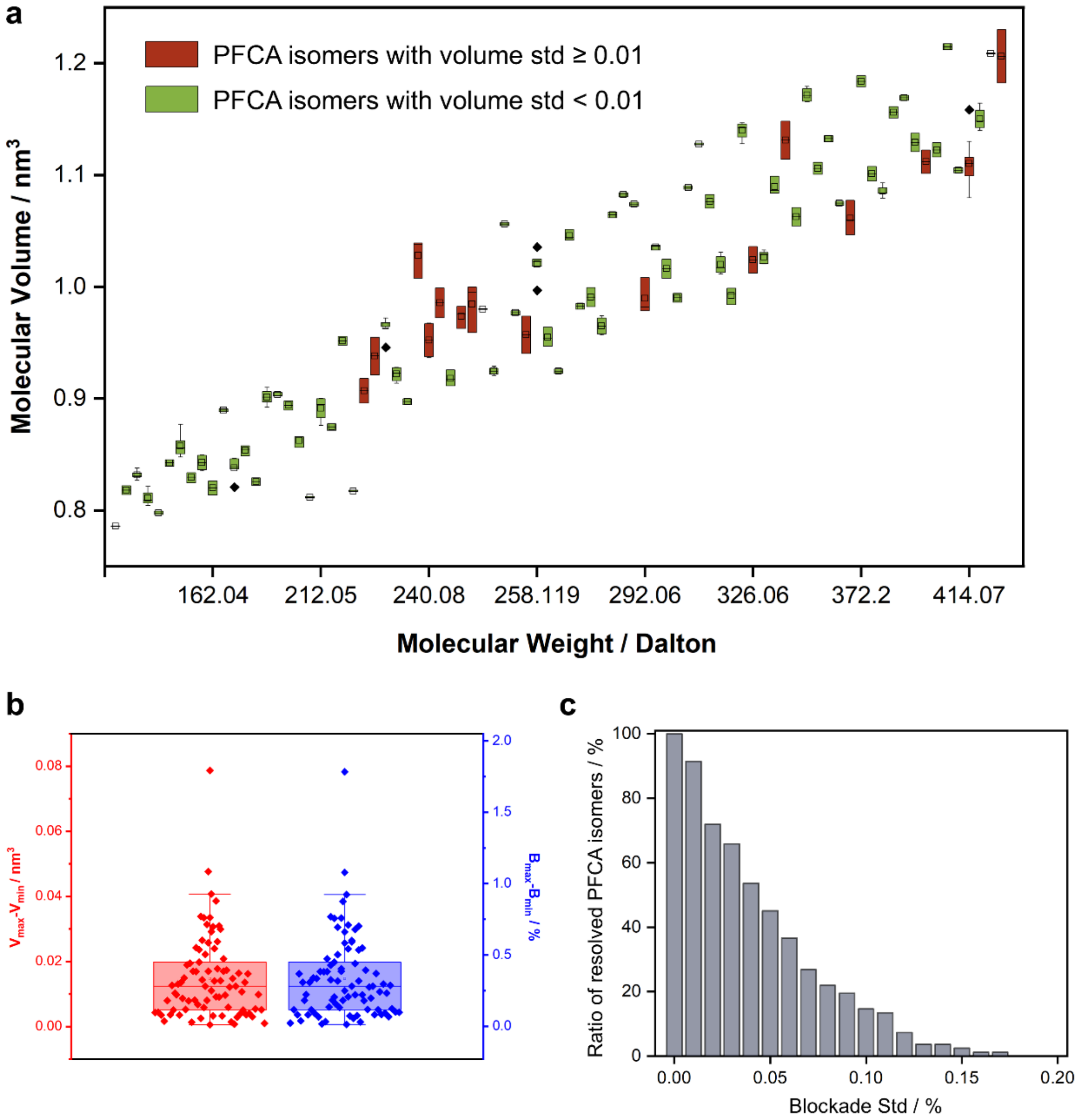

Supplementary Fig. 49. (a) Calculated molecular volumes for 83 sets of PFCA isomers (265 structures in total) with molar mass less than 500 Da. (b) Distribution of volume and blockade differences of PFCA isomers (83 sets in total). The center line, limits, whiskers, hollow point in box-plot (a, b) were respectively median value, 25th and 75th percentile, 1.5 times outliers, and mean value. The solid points in box-plot (b) were simulated volume differences and predicted blockade differences of PFCA isomers. (c) Percentage of fully discriminated PFCA isomers compared to the standard deviation of current blockades.

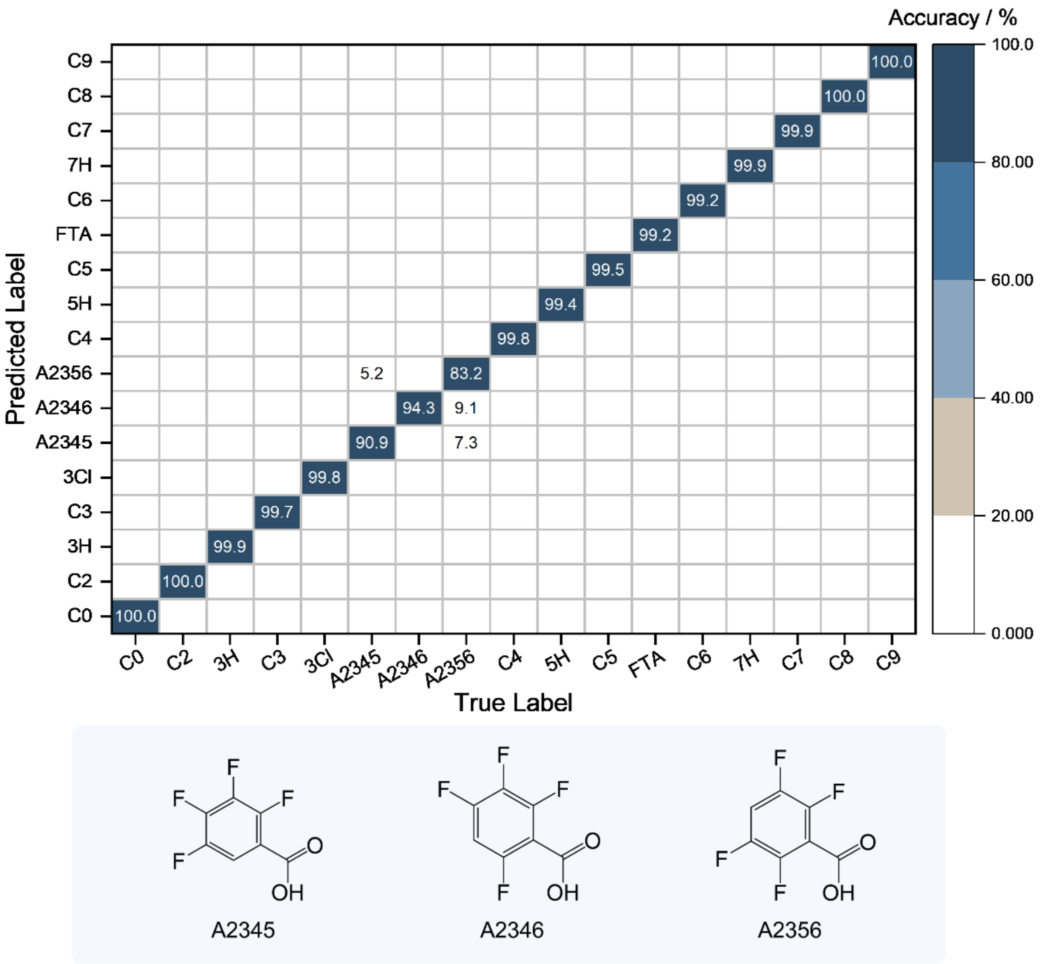

Supplementary Fig. 50. The confusion matrix for identification of 13 straight-chain PFCAs, 3 aromatic PFCA isomers (mono-hydrogen substituted) and R_6 probe by WT AeL nanopore, at 20°C and -50 mV.

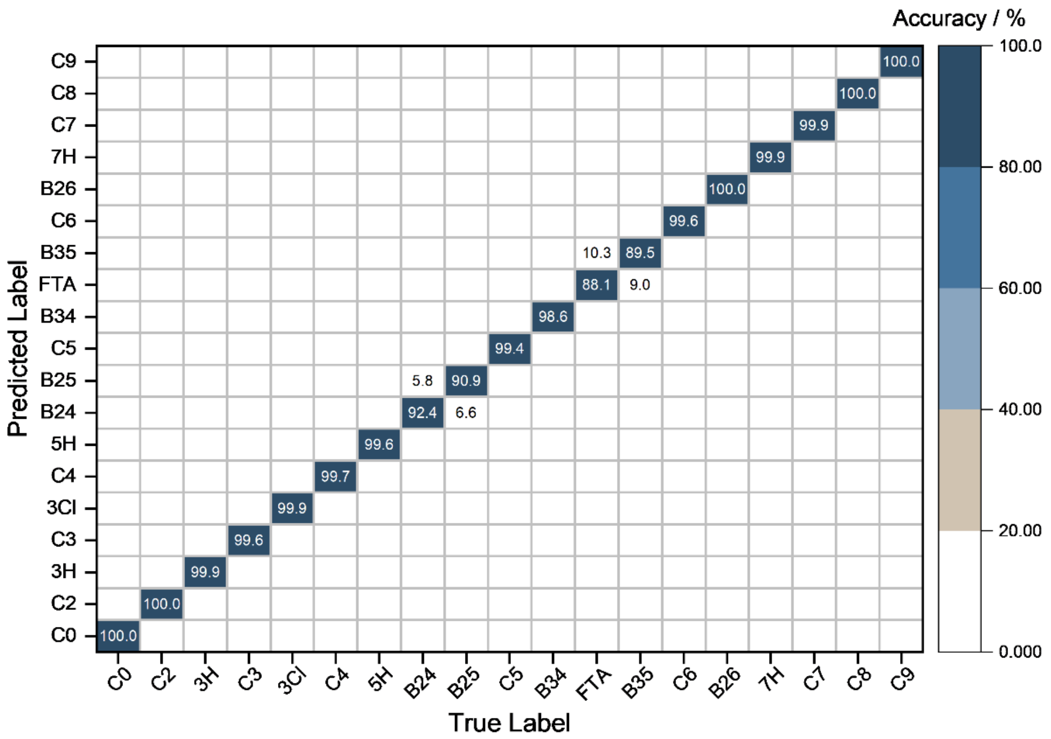

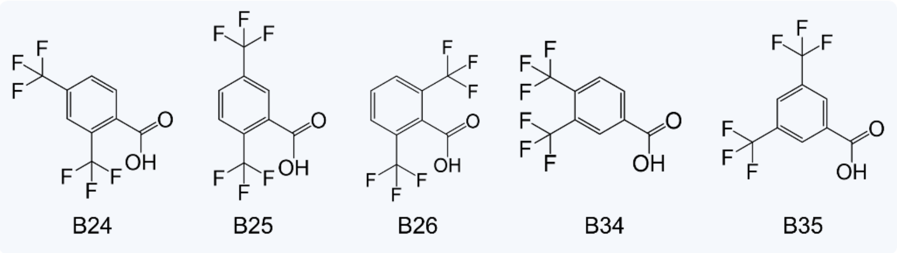

Supplementary Fig. 51. The confusion matrix for identification of 13 straight-chain PFCA, 5 aromatic PFCA isomers (tri-hydrogen substituted) and R_6 probe by WT AeL nanopore, at 20°C and -50 mV.

iv Reliability of the interval time calculation

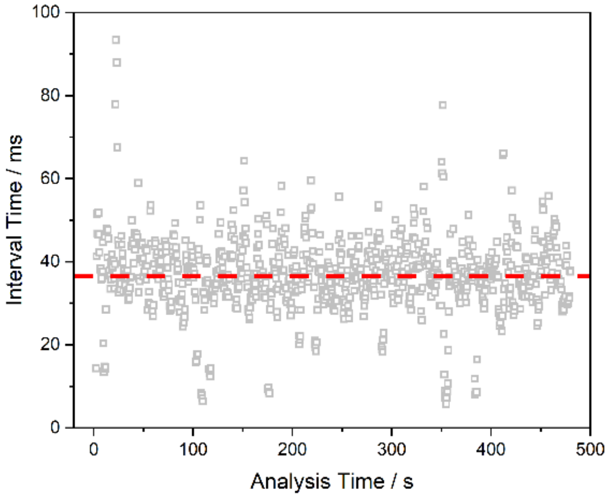

Supplementary Fig. 52. Interval time calculated at different analysis time. The red line denoted the average interval time of 36.7 ms over the full analysis time.

v Incubation of PFCAs with R_6 probes

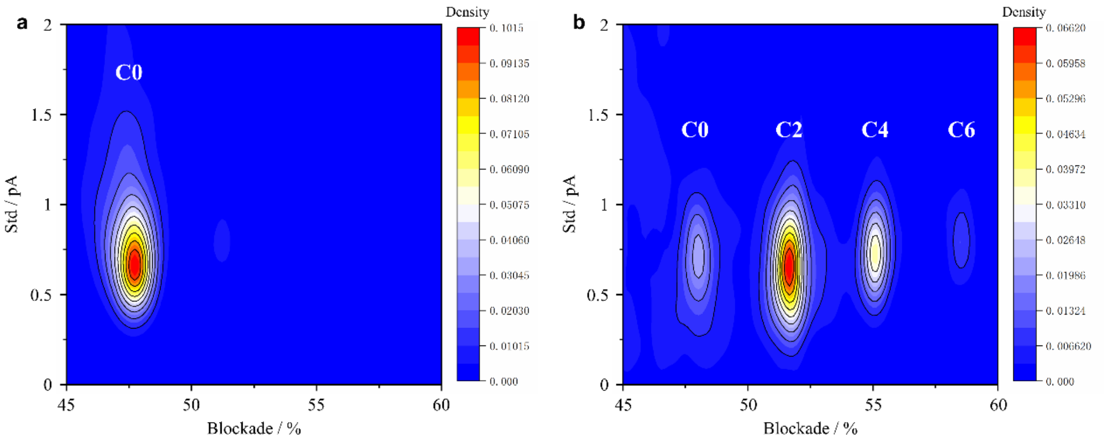

Supplementary Fig. 53. Typical blockade and standard deviation signals for (a) the R_6 probes (C0) and (b) the incubation of R_6 probes with different concentrations of activated C2/C4/C6. The overall duration for activation and incubation was roughly 1 hour.

VI Supplementary references

1. Gu, L.-Q. et al. Stochastic sensing of organic analytes by a pore-forming protein containing a molecular adapter. *Nature* **398**, 686-690 (1999).
2. Braha, O. et al. Simultaneous stochastic sensing of divalent metal ions. *Nat. Biotechnol.***18**, 1005-1007 (2000).
3. Austin, C. et al. Hydrothermal destruction and defluorination of trifluoroacetic acid (TFA). *Environ. Sci. Technol.* **58**, 8076-8085 (2024).
4. Cahill. T. M. Assessment of potential accumulation of trifluoroacetate in terminal lakes. *Environ. Sci. Technol.* **58**, 2966-2972 (2024).
5. Zheng, G., Eick, S. M., & Salamova, A. Elevated levels of ultrashort-and short-chain perfluoroalkyl acids in US homes and people. *Environ. Sci. Technol.* **57**, 15782-15793 (2023).
6. Sadia, M. et al. Occurrence, fate, and related health risks of PFAS in raw and produced drinking water. *Environ. Sci. Technol.* **57**, 3062-3074 (2023).
7. Zhao, M. et al. Nontarget identification of novel per-and polyfluoroalkyl substances (PFAS) in soils from an oil refinery in southwestern China: a combined approach with TOP assay. *Environ. Sci. Technol.* **57**, 20194-20205 (2023).
8. Dong, B. et al. Trace analysis method based on UPLC-MS/MS for the determination of (C2-C18) per-and polyfluoroalkyl substances and its application to tap water and bottled water. *Anal. Chem.* **95**, 695-702 (2023).
9. Cahill, T. M. Increases in trifluoroacetate concentrations in surface waters over two decades. *Environ. Sci. Technol.* **56**, 9428-9434 (2022).
